# Comparative analysis of thoracic and abdominal aortic aneurysms across the segment and species at the single-cell level

**DOI:** 10.3389/fphar.2022.1095757

**Published:** 2023-01-10

**Authors:** Hong Wu, Cheng Xie, Ruilin Wang, Jun Cheng, Qingbo Xu, Haige Zhao

**Affiliations:** ^1^ Department of Cardiology, The First Affiliated Hospital, Zhejiang University School of Medicine, Hangzhou, China; ^2^ Key Laboratory of Medical Electrophysiology of Ministry of Education and Medical Electrophysiological Key Laboratory of Sichuan Province, Collaborative Innovation Center for Prevention and Treatment of Cardiovascular Disease, Institute of Cardiovascular Research, Public Center of Experimental Technology, Southwest Medical University, Luzhou, China; ^3^ Department of Cardiovascular Surgery, the First Affiliated Hospital, Zhejiang University School of Medicine, Hangzhou, China

**Keywords:** thoracic aortic aneurysm, abdominal aortic aneurysm, species homology and diversity, single-cell RNA sequencing, comparative analysis

## Abstract

**Introduction:** Aortic aneurysm is a life-threatening disease resulted from progressive dilatation of the aorta, which can be subdivided into thoracic and abdominal aortic aneurysms. Sustained subcutaneous angiotensin II infusion can induce aortic aneurysms in mice. However, the relevance of using angiotensin II induction model to study aneurysm disease and the degree of commonality between species remain elusive.

**Methods:** We utilized scRNA-seq to infer aortic cell sub-structures and transcriptional profiles in clinical patient TAAs and AAAs, as well as mouse models of corresponding diseases (Ang II induction) and in healthy mouse aorta. Unbiased comparison between mice and humans explored the possible reasonability and utility of mouse Ang II-induced aortic aneurysm as a model for human aortic aneurysm diseases. Meanwhile, we performed comparative analysis of aortic aneurysms between TAA and AAA in both organisms.

**Results and Discussion:** We demonstrated similarities and differences of changes in the components of human and mouse cell types, and our unbiased comparison between mouse and human identified well conserved subpopulations of SMCs and macrophages. Furthermore, the results of our comparative analyses suggested different biological functions and distinct potential pathogenic genes for thoracic and abdominal aortic aneurysms. MIF and SPP1 signaling networks participated in aortic aneurysm in both organisms. This study maps aortic aneurysm and offers opportunities for future researches to investigate the potential of subpopulations or marker genes as therapy targets.

## Introduction

Aortic aneurysm is a life-threatening disease that results from progressive dilatation of the aorta and weakness of the blood vessel walls (Mizrak et al., 2022), which usually goes unnoticed until incidental detection during imaging examination or until rupture of the aorta, a disastrous event with a mortality rate of up to 50%–80% (Kuivaniemi et al., 2015). On the basis of anatomic locations, aortic aneurysms are subdivided into two categories: the thoracic aortic aneurysm (TAA, which occurs above the diaphragm) and abdominal aortic aneurysm (AAA, which occurs below the diaphragm). Although the two diseases share several etiological similarities, such as inflammation, proteolytic elastic tissue, degeneration of the extracellular matrix (ECM), and smooth muscle cell (SMC) apoptosis, there are also significant differences in the population prevalence rate, genetic patterns, and susceptibility genes. AAAs are more common than TAAs because they affect 4%–7% males over the age of 65 and 1%–2% in females (Lederle, 2003), whereas the prevalence of TAA is estimated to be around 1% of the general population (Verstraeten et al., 2017). In addition, approximately 20%–25% of TAA patients are estimated to have familial TAAs, and considerable proportion (∼25%) of the familial cases exhibit an autosomal dominant pattern of inheritance of the disease within the family, i.e., thoracic aortic disease caused by a mutation in a single gene (Marfan syndrome due to *FBN1* mutations, *COL3A1* mutations related to vascular Ehlers–Danlos syndrome) (Pinard et al., 2019), whereas AAA does not typically demonstrate such inheritance. Nonetheless, studies discover the involvement of genetic determinants linked to AAAs and identified several risk genes such as *AGTR1* and *MMP3*. Importantly, risk factors for AAA also include manageable smoking, hypertension, and hyperlipidemia (Vardulaki et al., 2000). The human aortic wall is composed of a variety of cell types and exhibits significant regional heterogeneity corresponding to their differing embryology (Cheung et al., 2012; Sawada et al., 2017), which greatly restricts our better knowledge of the pathogenesis of TAAs and AAAs. However, both TAAs and AAAs are complex, multifactorial, and highly heterogeneous diseases. An improved knowledge of the cellular mechanism networks that trigger the development and succeeding expansion of aneurysms is critical to discovering innovative therapeutic targets.

The second primary challenge in investigating aortic aneurysms is the potential discrepancy between mice and humans. The mouse remains the dominant research model for studying disease pathogenesis, partly not only because of their scalability and reproducibility compared to human investigations but also due to their utility value in the study of mechanism regulatory networks (Zilionis et al., 2019). Three experimental techniques, CaCl_2_ application, elastase perfusion, and angiotensin II (Ang II) infusion, can induce aortic aneurysms in mice (Lu and Daugherty, 2017; Sénémaud et al., 2017). The Ang II subcutaneous perfusion model has advantages of simulating several features of human aortic aneurysms, such as medial degeneration, thrombosis, accompanying with atherosclerosis, ECM degradation, endothelial permeability, and phagocyte and inflammatory cell infiltration (Sénémaud et al., 2017). Importantly, mouse Ang II-induced aneurysms generally occur in the ascending or the suprarenal aorta, whereas aortic aneurysms in humans usually take place in the infrarenal aortic segment (Sénémaud et al., 2017). The differences have aroused practical concerns and interests in the different gene expressions and pathological processes between mice and humans; therefore, it becomes crucial to link population structures of humans and mice.

To address both difficulties, single-cell RNA sequencing (scRNA-seq) provides an opportunity to sample the entire transcriptome of a single cell, thus clustering similar cells independently of any previous assumptions on cellular marker genes or species conservation and allowing for unbiased comparisons between organisms (Briggs et al., 2018). Here, we used scRNA-seq to infer aortic cell sub-structures and transcriptional profiles in clinical patient TAAs and AAAs, as well as mouse models of corresponding diseases (Ang II induction), and in healthy mouse aorta. Unbiased comparison between mice and humans explored the possible reasonability and utility of mouse Ang II-induced aortic aneurysm as a model for human aortic aneurysm diseases. Meanwhile, comparative analysis of aortic aneurysms of different cross sections revealed distinct pathogenic genes between TAAs and AAAs in both organisms. Our findings discovered commonalities in the pathogenesis and gene expression profiles of aortic aneurysms between mice and humans, opening up the possibility of using specific sub-populations as potential therapeutic targets and exploring them in mice.

## Materials and methods

### Collection of human samples for histological analysis

The protocol for collecting human tissue samples was approved by the Research Ethics Committees of the First Affiliated Hospital of Zhejiang University School of Medicine. Normal ascending aortic tissue samples were collected from recipients of heart transplants or lung donors, and diseased thoracic aortic tissue specimens were collected from patients with sporadic TAAs; one abdominal aneurysmal aortic tissue was collected from patients with AAA. These tissues were collected for histopathological analysis. Clinical characteristics of the study groups of AAA and TAA are summarized in Supplementary Dataset S1.

### Animal and ethics statement

All animal procedures were in accordance with the Guide for Care and Use of Laboratory Animals published by the US National Institute of Health (8th edition, 2011) and were approved by the Institutional Animal Care and Use Committee of the Zhejiang University School of Medicine. ApoE^−/−^ mice were purchased from the Shanghai Model Organisms Center at the age of 8 weeks. Here, 12-week male ApoE^−/−^ mice were continuously treated with subcutaneous Ang II infusion for 4 weeks (1,000 ng/kg/min, MCE, Cat. No.: HY-13948) by using an osmotic pump (Alzet model 1004, ALZA Corporation).

### Immunofluorescence staining

Human aortic tissue specimens were dehydrated, embedded, and cut into 3-μm-thick sections using a Leica RM2235 manual rotary microtome. Tissue sections were deparaffinized and hydrated in gradient xylene and ethanol. The antigen retrieval solution used was prepared with 10 mM Tris, 1 mM EDTA, and .05% Tween 20 (pH 9.0) by placing in 98°C water bath for 25 mins. After cooling down to room temperature (RT), tissue was blocked and permeabilized with 5% BSA+.1% Triton X-100 (Sigma, T8787) for 1 h at RT, stained with primary antibodies overnight at 4°C, then incubated with Alexa Fluor-conjugated secondary antibodies (1:500, Invitrogen) for 1 hour, and stained with DAPI (Servicebio, G1012).

Immunofluorescence staining of frozen sections from mouse aortic artery specimens was performed, arteries were first harvested, washed in PBS and fixed in 4% paraformaldehyde for 2 h at 4°C, and then dehydrated at 4°C in 30% sucrose solution overnight until fully penetrated. Tissues were then embedded in optimal temperature (O.C.T., Sakura, 4583) and cut into 8-µm sections by a cryostat (Leica CM1950). The frozen sections were air-dried for about 30 mins at room temperature, permeabilized and blocked in 5% donkey serum (Solarbio® 41, SL050) for 1 h, incubated with primary antibodies at 4°C overnight, and then stained with Alexa Fluor-conjugated secondary antibodies (1:500, Invitrogen) for 1 hour, followed by DAPI (Servicebio, G1012) staining.

Primary antibodies were used as listed: SMA-FITC (1:500, Sigma, F3777), SM22 alpha (1:200, Abcam, ab14106), SM-MHC (1:200, Abcam, ab53219), CD45 (1:50, R&D, AF114), CD68 (Abcam, ab125212, 1:200), and vimentin (Abcam, ab8978, 1:200). Secondary antibodies were used as listed: donkey anti-rabbit IgG Alexa Fluor 555 (Invitrogen, A-31572, 1:500), donkey anti-rat IgG Alexa Fluor 488 (Invitrogen, A-21208, 1:500), donkey anti-mouse IgG Alexa Fluor 488 (1:500, Invitrogen, A-21202), donkey anti-goat IgG Alexa Fluor 488 (Invitrogen, A-11055, 1:500), and donkey anti-goat IgG Alexa Fluor 647 (1:500, Invitrogen, A-21447) antibodies. Isotype control primary antibodies (Invitrogen, Cat No. 31933, 02-6102, 31903, and 31245) for each host species were used as negative controls, together with secondary antibodies, and only control to validate specificity of antibodies and to eliminate the background signal. The images were taken with a Leica SP5 confocal microscope and analyzed with LAS AS software (Leica).

### Isolation of single cells from the aorta

We digested the aortic vessels according to the method reported by our group (Deng et al., 2020; Jiang et al., 2021). Mice were euthanized and perfused with PBS, and aortic arteries were harvested and placed in a Petri dish containing DMEM (ATCC, 302002) with 10% fetal bovine serum (FBS, Gibco, 10099141) on ice. The perivascular connective tissue and adipose tissue were carefully removed. After collecting all arteries, arteries were washed with PBS, cut into pieces, and digested with 0.75 mg/ml papain digesting solution. After 1–2 digestions, the rest tissues were digested with 0.75 mg/ml papain and 1 mg/ml collagenase I in Hank’s solution (HBSS, Gibco, 14025092). The whole process of digestion was carried out in 37 water baths with reciprocating shaking. The detached cells were collected in DMEM containing 20% FBS, and fresh digestion solutions were replaced every 10 mins until the tissues were digested completely. At the end of the digestion process, all the cells digested from one sample were collected together. After thorough digestion, we filtered the cells with a 40-μm cell filter and centrifuged at 500 g speed at 4°C for 8 mins. Subsequently, the cell pellet was resuspended in relevant solutions for further experiments.

### Flow cytometric analyses

For isolated aortic single cells, the harvested cells were incubated with red blood cell lysis buffer, and then, single-cell suspensions were obtained using a 40-μm cell strainer. The cells were stained with conjugated antibodies (1 μg per 10,634 cells) for 30 min at 4°C. Conjugated antibodies used include CD45-FITC (Invitrogen, 11-0451-82), F4/80-PE-Cyanine7 (Invitrogen, 25-4801-82), CD11B-PE (Invitrogen, 12-0112-82), LY6C-APC (17-5932-82), and CD3-PE (Invitrogen, 12-0031-82). An Alexa Fluor® 38488 conjugated rat IgG2a, κ isotype control (BD Pharmingen™, 557676) was used to validate specificity of antibodies. Then, the cells were washed and suspended and stained with a LIVE/DEAD™ Fixable Near-IR Dead Cell Stain Kit (1:1000, Invitrogen, L34975) for 5 mins. The cells were then washed and re-suspended in PBS with 1% FBS for FACS analyses. All prepared samples were analyzed by using a BD LSR Fortessa II flow cytometer (BD Biosciences). FlowJo v10 software (BD Biosciences, United States) was used to analyze the flow cytometric data.

### ScRNA-seq of artery cells with 10× chromium and data analysis

Human scRNA-seq datasets for TAA (GSE155468) and AAA (GSE166676) were collected from public repositories. We performed scRNA-seq of thoracic and abdominal aorta cells, respectively, from Ang II-induced mice and the control group. After full digestion, the cells were suspended with PBS and stained with a LIVE/DEAD™ Fixable Near-IR Dead Cell Stain Kit and Hoechst 33342 (1:1000, Invitrogen, H3570) for 20 mins on ice. We then sorted single nucleated live cells into PBS containing .04% BSA by BD FACS ARIA II flow cytometry (BD Biosciences). The samples were subjected to scRNA-seq using the Chromium™ Single Cell 3′ Reagent Kit v2 or v3 chemistry (10x Genomics) and followed a standard protocol. The library was generated and sequenced on a NovaSeq 6000 PE150 platform (Illumina) with the paired-end 150-bp sequencing strategy. The 10x Chromium^TM^ procedure, library generation, and sequencing were carried out by Novogene Co., Ltd. (Beijing, China).

Cell filtration, data normalization, dataset integration, and further cell clustering and visualization were performed with the R package Seurat (version 4.0.1) with default parameters, unless otherwise specified (Stuart et al., 2019). Briefly, gene signatures expressed in cells with fewer than three cells and cells expressing fewer than 100 or greater than 4,000 genes were excluded for filtering out non-cell or cell aggregates. Moreover, the cells expressing mitochondrial gene percentages greater than 5% were also excluded. Then, the DoubletFinder R package was performed to filter out doublet cells (McGinnis et al., 2019). After alignment and quality control, 8,297 cells from non-diseased thoracic aortic wall tissue (normal TA, *n* = 3), 38,681 cells from aneurysmal thoracic aortic wall tissue (TAA, *n* = 8), 4,815 cells from the normal abdominal aorta group (normal AA, *n* = 2), and 7,257 cells from the abdominal aortic aneurysm group (AAA, *n* = 4) were combined and included in the subsequent analysis; mouse Ang II-induced thoracic aorta (11,438 cells, *n* = 2 samples), sham thoracic aorta (5,187 cells), Ang II-induced abdominal aneurysm (13,102 cells, *n* = 2 samples), and sham abdominal aorta (5,804 cells) were aggregated and included in the subsequent analysis. After log normalization, the top 2,000 highly variable genes were chosen and scaled by “ScaleData.” Datasets were integrated in Seurat using first 30 dimensions. The dimension was then reduced with 30 principal components, and the t-distributed stochastic neighbor embedding (t-SNE) was used to visualize clusters with the resolution set at .5.

### Gene enrichment analyses

Gene ontology and KEGG pathway analyses of DEGs were performed using the R package clusterProfiler (https://github.com/YuLab-SMU/clusterProfiler) (Yu et al., 2012). The biological process of gene ontology was annotated by the enrichGO function with annotation databases org.Hs.eg.db and org.Mm.eg.db. Biological process expression levels referred to the calculation of the average expression of gene sets in BP annotation using the online database (geneontology.org) and AddModuleScore in the Seurat R package.

### T-cell score definition

Genes related to CD4/CD8 T-cell resident markers, exhausted markers, cytotoxic markers, and co-stimulatory score markers are from published studies (Guo et al., 2018; Zhang et al., 2020). The average expression levels of these gene sets were used to determine immune states for T cells.

### Pseudotime trajectory analyses

Pseudotime trajectory analyses were conducted with the R package Monocle (version 2.14) with default settings. The top 250 (arranging by avg log2 fold change) differentially expressed genes (DEGs) were used for pseudotime by the function differentialGeneTest with fullModelFormulaStri set as pseudotime. For dimension reduction and subsequent cell ordering along the pseudotime trajectory, the DDRTree method was used.

### Cell–cell communication

CellChatDB is a literature-supported database of mouse and human ligand–receptor interactions (Jin et al., 2021). We eliminated cycling cells and unspecific cells for cell–cell communication analysis. Then, Seurat-preprocessed human and mouse datasets were separately loaded to the CellChat package to analyze and visualize the cell–cell interaction. The interesting network pathways among source cells and target cells were selected and visualized by the hierarchy plot. Comparison of contribution of significant ligand–receptor pairs in the signaling between indicated cell types among each group was analyzed and displayed by the dot plot.

### Data availability

The data and R scripts that linked to the results of the study are available on reasonable request. ScRNA-seq data on the study are available in the Gene Expression Omnibus dataset. The scRNA-seq datasets for human TAAs (GSE155468) and AAAs (GSE166676) were collected from public repositories.

### Statistics

All statistical analyses and presentations were conducted with the R package ggsignif. Comparisons of the expression levels of gene sets or gene signatures between the two groups were carried out by Wilcoxon rank-sum tests. Statistical tests used in figures are shown in corresponding figure legends. The precise value of n is displayed in the figure legends, and the meaning of n is also displayed in the figure legends.

## Results

### scRNA-seq delineated cellular landscapes in mouse and human aortic aneurysms

**FIGURE 1:**
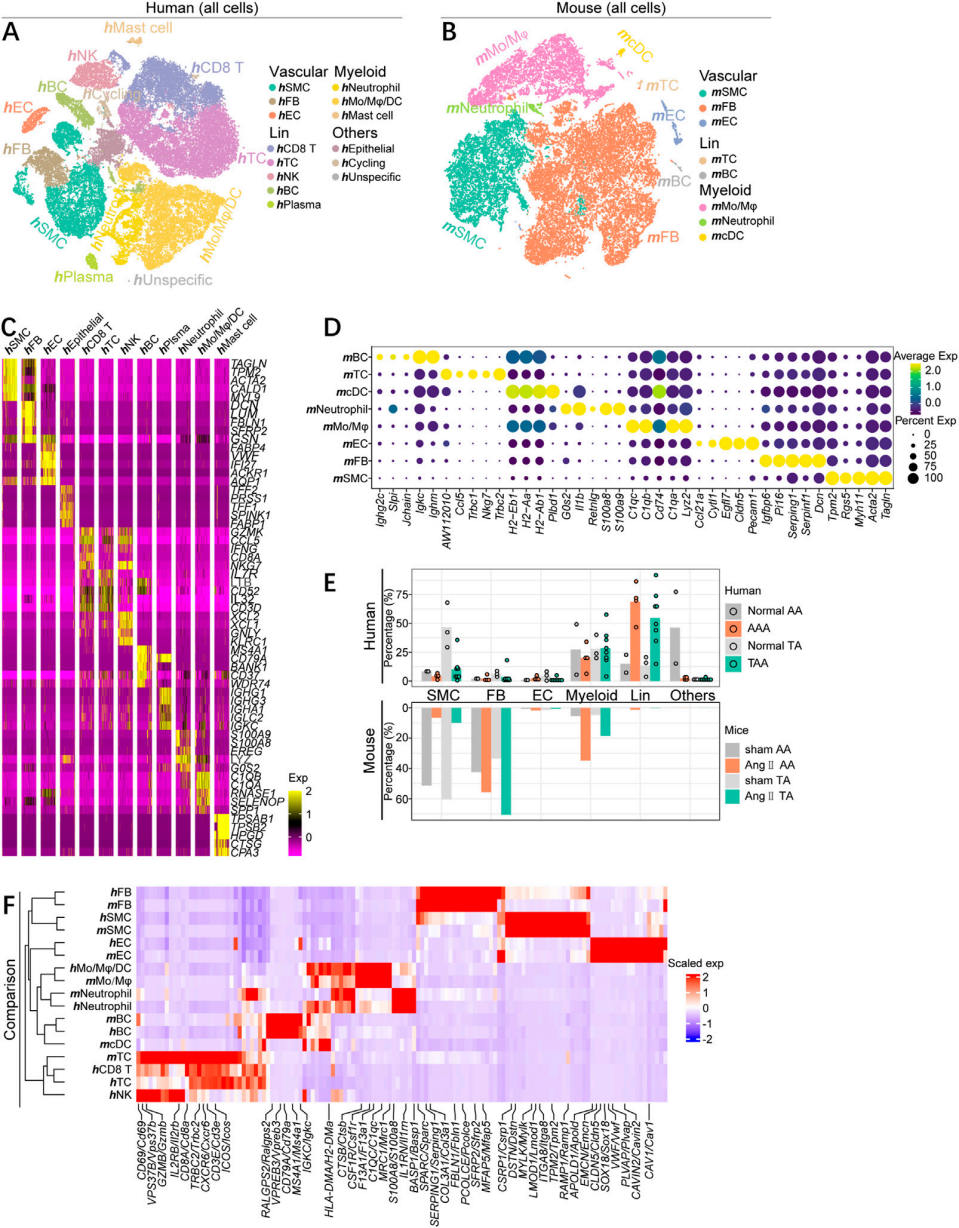
scRNA-seq profiling map of cell gene expression in human and mouse aortic aneurysms. **(A)** T-distributed stochastic neighbor embedding (t-SNE) plot, showing the annotation and color codes for major cell types of all aorta cells from human TAA patients (38,681 cells, *n* = 8 patients) and normal thoracic aorta (8,297 cells, *n* = 3 patients), and AAA patients (7,257 cells, *n* = 4 patients), and normal abdominal aorta (4,815 cells, *n* = 2). Major cell types were defined by canonical lineage markers. **(B)** t-SNE plot, showing the annotation and color codes for major cell types of all aorta cells from mouse Ang II-induced thoracic aorta (11,438 cells, *n* = 2 samples, six mice in each sample) and sham thoracic aorta (5,187 cells, *n* = 2 samples, seven mice in each sample), and Ang II-induced abdominal aneurysm (13,102 cells, *n* = 2 samples, six mice in each sample) and sham abdominal aorta (5,804 cells, *n* = 2 samples, seven mice in each sample). **(C)** Heatmap showing the expression of marker genes in each cell types of human aorta cells. **(D)**Dot plot is displaying average scaled expression levels (color scaled, column-wise Z scores) of top DEGs (columns) across mouse major cell types. The circle size indicates the cell fraction expressing signatures greater than mean; color indicates mean signature expression (yellow, high; blue, low). **(E)** Fractions of major cell types in each dataset of aneurysmal aorta and corresponding normal aorta of humans and mice. **(F)** Heatmap showing gene orthologs similarly enriched within mouse and human major cell types. Orthologous mouse and human major cell types are established by hierarchical clustering. Heatmap showing genes similarly enriched within mouse and human major cell types. The gene set shown is the intersection of top 50 most enriched genes per cell type between mice and humans. Scaled exp, scaled expression value per gene.

Our study set out to determine aortic aneurysm cell sub-clusters from human TAAs and AAAs and in a mouse Ang II-induced aortic aneurysm model and to find homologies and discrepancies across aortic segments and organisms. We respectively collected human scRNA-seq datasets for TAAs (GSE155468) and AAAs (GSE166676) from public repositories (Li et al., 2020; Davis et al., 2021). After alignment and quality control, 8,297 cells from non-diseased thoracic aortic wall tissue (Normal TA, *n* = 3), 38,681 cells aneurysmal thoracic aortic wall tissue (TAA, *n* = 8), 4,815 cells from the normal abdominal aorta group (Normal AA, *n* = 2), and 7,257 cells from the abdominal aortic aneurysm group (AAA, *n* = 4) were combined and included in the subsequent analysis. The population structure of these human cells was analyzed and annotated according to acknowledged molecular markers, as shown in Supplementary Figures S1A–C. All cell types were classified as vascular wall cells [vascular smooth muscle cells (***h*** SMCs), endothelial cells (***h*** ECs), and fibroblast (***h*** FBs)], myeloid immune cells [***h*** Neutrophils, monocytes/macrophages/dendritic cells (***h*** Mo/Mφ/DC), and ***h*** mast cells], lymphocytes [***h*** T cell, ***h*** *CD8* ^+^ T cells, natural killer cells (***h*** NK), ***h*** B cells, and ***h*** plasma cells], and others (***h*** epithelial cells, ***h*** cycling cells, and ***h*** unspecific cells) (Figure 1A).

The matched dataset of mouse cells was acquired from the Ang II-induced aneurysmal abdominal aorta (Ang II AA) and thoracic aorta (Ang II TA), as well as the corresponding sham group (sham AA and sham TA). Mouse cells were partitioned with t-SNE into 22 clusters and were identified as eight major cell types *via* the expression of canonical lineage markers (Supplementary Figures S1D, E). Similarly, all mouse cells consisted of three major classifications, including vascular wall cells (***m*** SMC, ***m*** EC, and ***m*** FB), myeloid immune cells (***m*** Mo/Mφ, ***m*** Neutrophil, and ***m*** cDC), and lymphocytes (***m*** TC and ***m*** BC) (Figure 1B), each containing cells from all four groups (Supplementary Figure S1F).

Both mouse and human cells were divided into major cell types, some of which contained complex subpopulation structures. To further uncover the characteristics of the cells, we identified differentially expressed genes (DEGs) of each cell clusters and displayed top five DEGs arranged by log2 fold change (Figures 1C, D). Albeit each cell type identified here possesses sub-cluster, the gene expression patterns annotated on the basis of acknowledged gene signatures reflected many known markers. Regardless of humans or mice, thoracic or abdominal aorta, the aortic SMC percentage was significantly lower than that of the corresponding normal group, as confirmed by quantification of the SMC composition of each sample. Fibroblasts apparently expanded in different segments of mouse aneurysmal aorta, while the proportion of fibroblasts in the human aorta was low and did not alter much in the diseased state. Moreover, the increase of inflammatory cells was predominantly lymphocytes in human aortic aneurysm specimens, whereas the enhancement of myeloid immune cells was dominated in mice (Figure 1E). To comprehensively compare the gene expression profiles of mouse and human cells, we quantified the homologies between average transcriptome of cell types recognized in the two organisms by visualizing the intersection of top 50 DEGs. We observed that the consistency of cell types, rather than source species, determined the homologies of gene expression patterns, as indicated by examining the expression of unique cell type genes in both mice and humans, as well as by hierarchical clustering tree of the major cell lineages. Except for ***m*** cDCs, every major cell type clustered with its homolog (Figure 1F). However, the gene expression profiles of ***m*** cDCs were more similar to neutrophils and Mo/Mφ/DCs. An extensive conservation of primary gene expression profiles in aortic cells was observed through unbiased comparison of mouse and human cells.

Collectively, there are both differences and similarities in the composition of cellular components and the changes in the aortic aneurysm disease status across segments and species. More importantly, this cell type correspondence reaffirms that the existence of consistent gene expression patterns between mice and humans and justifies an inspection of similarities and discrepancies within each cell lineage.

### Alteration of cellular composition in the diseased status of mice and humans

**FIGURE 2:**
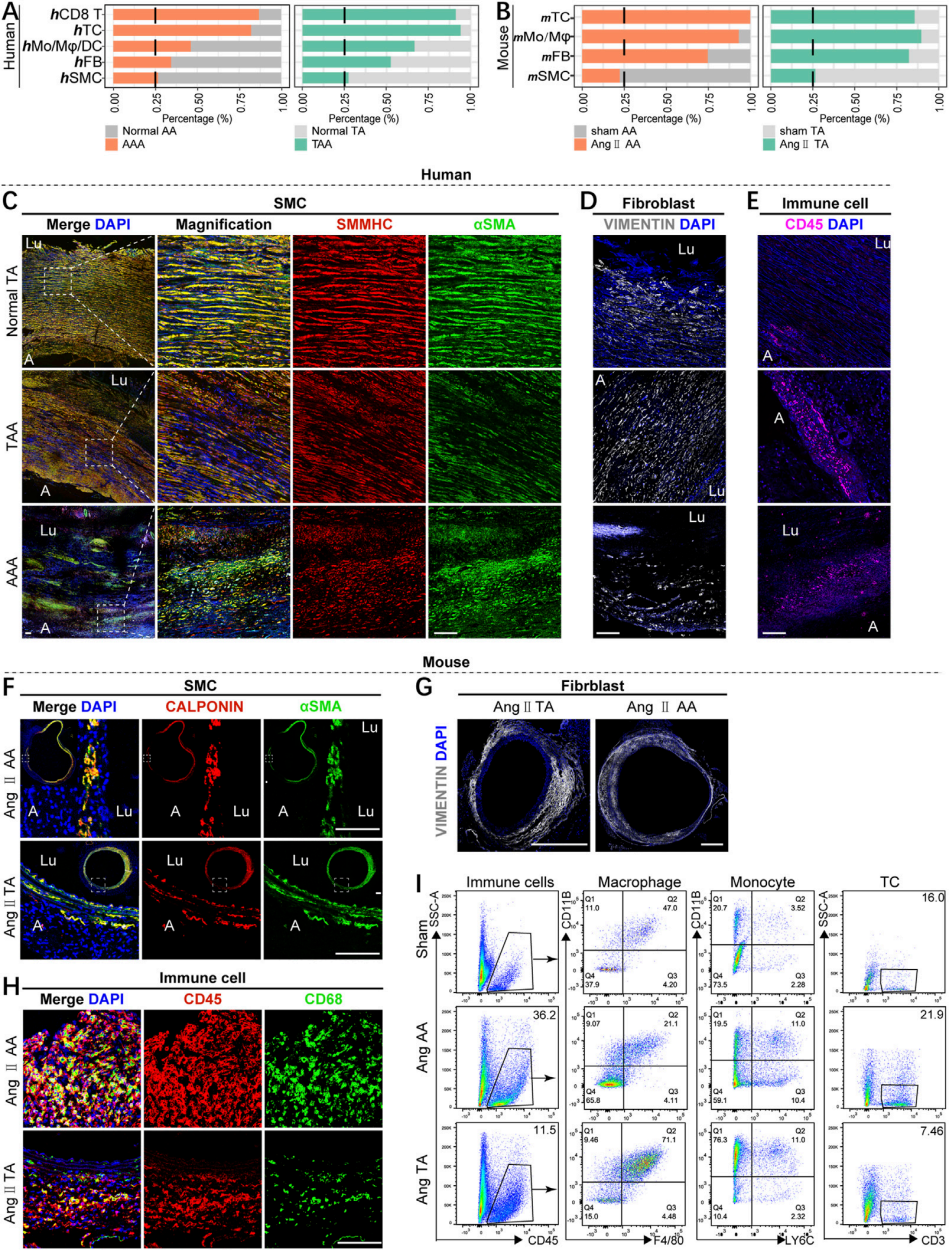
Alteration of cellular composition in the diseased status of mice and humans. **(A,B)** Fractions of major cells types in aneurysmal aorta *vs.* the corresponding normal aorta of humans **(A)** and mice **(B)**. **(C)** Immunofluorescence analysis for SMMHC (red), αSMA (green), and overlays with DAPI-labeled nuclei (blue) showing smooth muscle cells in the human vascular media (scale bars: 100 μm). Lu means the lumen side of the vascular wall, and A means the adventitia side of the vascular wall. **(D,E)** Immunofluorescence analysis for vimentin (white) **(D)** and CD45 (purple) **(E)** and overlays with DAPI-labeled nuclei (blue) showing fibroblasts and immune cells in the human aorta (scale bars: 100 μm). **(F)** Immunostaining for calponin (red), αSMA (green), and overlays with DAPI-labeled nuclei (blue) showing smooth muscle cells in the mouse aortic media (scale bars: 100 μm). **(G)** Immunostaining for vimentin (white) and DAPI overlay in the thoracic and abdominal aorta form mice subjected to 4 weeks of Ang II induction (scale bars: 500 μm). **(H)** Immunostaining for CD45 (red), CD68 (green), and overlays with DAPI-labeled nuclei (blue) showing macrophages in the mouse aorta (scale bars: 100 μm). **(I)** Flow cytometry of the proportion of macrophages (F4/80^+^CD11B^+^), monocytes (LY6C^+^CD11B^+^), and T cells (CD3^+^) in CD45^+^ immune cells among three groups (n = 3–4, per group).

Our analysis revealed the remarkable reduction of SMC proportion and increase of immune cells including T cells and monocytes/macrophages (Mo/Mφ) in aneurysmal aorta compared with corresponding controls both in mice and humans, although the absolute number of T cells in the mouse aorta was very small; different from human aortic aneurysm, the number and percentage of mouse Mo/Mφ elevated significantly on the pathological condition (Figures 2A, B). To further validate our analysis, we collected clinical diseased aortic wall specimens from thoracic and abdominal aorta aneurysm patients and non-dilated ascending aortic wall tissues from lung donors. Histological analysis confirmed elastin degradation manifested by increased elastin fragmentation and even elastin loss, as well as collagen deposition, especially in focal areas with severe disruption of elastin (Supplementary Figure S2A). Immunofluorescence (IF) staining of the mature SMC marker SMMHC and the SMC contractile marker αSMA demonstrated a significant decrease of SMC density in aortic vascular media and phenotypic switches in abdominal aortic aneurysm indicated by SMC morphology alteration (Figure 2C). Moreover, the number of fibroblasts increased in thoracic aortic aneurysm patients, which might be inconsistent with scRNA-seq data, could be due to tissue digestion and individual differences; fibroblasts did not enhance in the AAA group (Figure 2D). Meanwhile, CD45^+^ inflammatory cells were obviously infiltrated in the diseased group (Figure 2E).

In line with humans, mice Ang II-induced aortic aneurysm displayed remarkable elastin break and reduction of number of SMCs (Figure 2F; Supplementary Figure S2B). However, different from human samples, mouse thoracic and abdominal aortic adventitia were markedly thickened and increase of fibroblast proportion, especially in Ang II-induced thoracic aorta (Figure 2G; Supplementary Figure S2B). In addition, IF staining indicated a lot of inflammatory cells and macrophages accumulated in the aortic wall after Ang II stimulation (Figure 2H). The infiltration of macrophages, monocytes, and T cells was verified by flow cytometry (Figure 2I). Generally, the Ang II-induced aortic aneurysm model showed similar pathological changes of SMCs and immune cells but different fibroblast alterations with clinical human thoracic and abdominal aortic aneurysm diseases.

### Aortic aneurysms contain well-conserved SMC subsets across species and distinct marker genes for TAAs and AAAs

**FIGURE 3:**
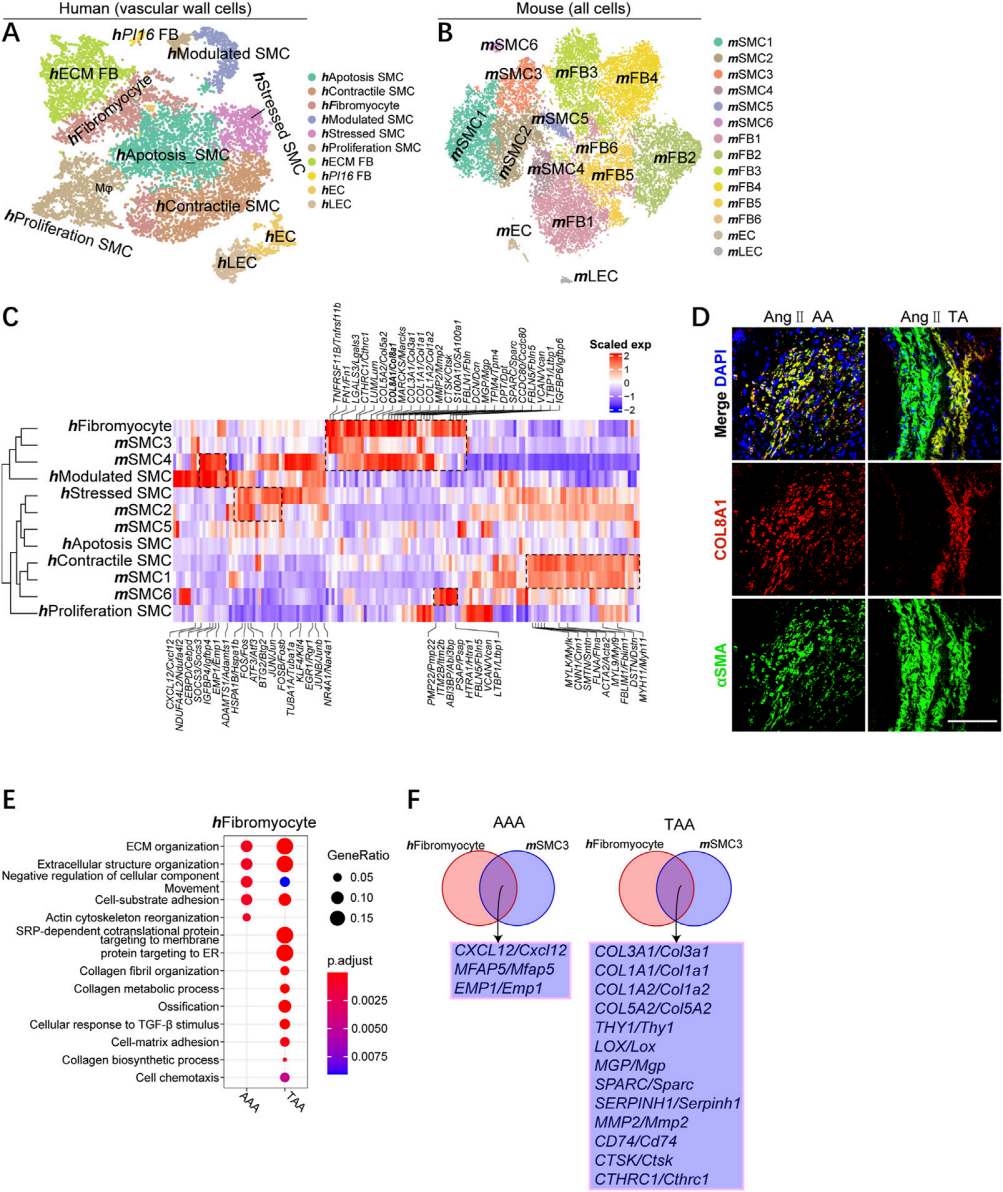
Identifying well-conserved SMC subsets and distinct marker genes for TAA and AAA across species. **(A)** T-SNE plot showing the annotation and color codes for human vascular wall cells. **(B)** t-SNE plot, clustering mouse aortic cells, showing cell clusters by color. **(C)** Comparison of mouse and human SMC subsets. Orthologous mouse and human SMC subsets established by hierarchical clustering. Heatmap showing genes similarly enriched within mouse and human SMC subsets. The gene set and scaled expression defined, as shown in Figure 1F. **(D)** Representative immunofluorescence images: COL8A1 (red), αSMA (green), and DAPI (blue), scale bar 100 μm. **(E)** Representative GO terms and pathways enriched in upregulated DEGs of ***h*** Fibromyocytes between TAA and AAA groups. TAA group: TAA vs. Normal TA; AAA group: AAA vs. Normal AA. **(F)** Integrated comparative analysis of upregulated DEGs of AAA (left) and TAA (right) between ***h*** Fibromyocytes and ***m*** SMC3s.

We then analyzed the sub-clusters of each classification, focusing on SMCs, macrophages, and T cells. Clustering human vascular wall cells identified six SMC subpopulations, two subset fibroblast subtypes, endothelial cells (ECs), and lymphatic endothelial cells (LECs), which were named based on previously reported human SMC subgroups and the corresponding functional enrichment analysis (Li et al., 2020; Mizrak et al., 2022) (Figure 3A; Supplementary Figures S3A–D). Clustering mouse vascular wall cells uncovered six ***m*** SMC sub-clusters, six ***m*** fibroblast subpopulations, ***m*** ECs, and ***m*** LECs (Figure 3B; Supplementary Figures S3A, C).

Unbiased comparison of mouse and human vascular wall cells also indicated the aggregation of the same cell type rather than species except for ***h*** fibromyocyte, ***h*** Modulated SMC, ***m*** SMC3, and ***m*** SMC4 (Supplementary Figure S4A). On the other hand, ***h*** fibromyocyte, ***h*** Modulated SMC, ***m*** SMC3, and ***m*** SMC4 were more similar to fibroblasts than SMC, reflecting their proximity in gene expression and had undergone phenotypic transformation into synthetic SMC (Supplementary Figure S4A). The percentage of ***h*** fibromyocyte and ***h*** Modulated SMC in human aneurysm and the proportion of ***m*** SMC3 and ***m*** SMC4 in mouse aortic aneurysm consistently enhanced in contrast to other subsets of SMCs (Supplementary Figure S4B).

To define how SMC subgroups associate with each other across organisms, we compared mouse and human SMC subtypes comprehensively on the entire transcriptome levels and the single-cell levels. Species conservation of several aspects of SMC subpopulation structures between humans and mice were observed, which could be demonstrated by an unsupervised comparison (Figure 3C). In both organisms, we observed highly similar subpopulations. ***h*** Contractile SMC and ***m*** SMC1 highly expressed plenty of canonical contractile SMC markers (*MYH11*, *DSTN*, *MYL9*, *CNN1*, and *MYLK*; omitting mouse gene symbols with synonymous lowercase here and later), whereas ***h*** Stressed and ***m*** SMC2 displayed high expression of stress response-related genes, such as *ATF3*, *FOS*, *JUN*, and *HSPA1B*, suggesting the stress response state in this type of SMC. ***m*** SMC6 was most closely associated with their ***m*** SMC1 counterparts (e.g., ***m*** SMC6 had low expression of contractile SMC markers), although ***m*** SMC6 uniquely expressed *Vcan* and *Ltbp1.* More importantly, ***m*** SMC3 are more similar to ***h*** Fibromyocyte than ***m*** SMC4 according to the hierarchical clustering tree, but ***m*** SMC4 also closely associated with these two subgroups because all three groups possessed the expression of classic synthetic SMC markers [collagen genes, such as *Col1a1*, *Col1a2*, and *Col3a1*; fibrosis-associated genes (*Fn1*, *Sparc*, and *Fbln*); and matrix metalloproteinase (MMP) gene (*Mmp2*)]. Meanwhile, we identified several innovative marker genes well-conserved across species, including *CTHRC1*, *COL8A1*, and *LGALS3*, involved in phenotypic switching into synthetic SMCs. In addition, we observed that the gene expression of ***m*** SMC4 resembled ***h*** Modulated SMC, and both of them highly expressed cytokines *CXCL12*, and *CEBPD* and *SOCS3*, further suggesting they were phenotypically transformed SMCs (Figure 3C). IF staining demonstrated the colocalization of COL8A1 and αSMA in phenotypic-transformed SMCs in mouse thoracic and abdominal aortic aneurysms, but COL8A1 was not expressed in contractile smooth muscle (Figure 3D; Supplementary Figure S4C ).

We next focused on ***h*** Fibromyocyte and ***m*** SMC3 subgroups due to their phenotypic transformation and their increase percentages among SMCs during the pathological process of aneurysm. By conducting comparative GO analysis, we studied the biological implication of TAA and AAA-related upregulated DEGs and found that both ***h*** Fibromyocyte and ***m*** SMC4 enriched for cell chemotaxis and cell–cell adhesion in the TAA group compared with AAA group; **m**SMC4 in TAA and AAA exhibited similar enrichment for collagen fibril organization, ossification, and cell adhesion (Figure 3E; Supplementary Figure S4D). In both organisms, we uncovered that *CXCL12*, *MFAP5*, and *EMP1* might participate in the pathogenesis of AAA, among which it had been reported that the blockade of CXCL12/CXCR4 protected against AAA formation (Michineau et al., 2014), whereas several collagen genes (*COL1A1*, *COL1A2*, *COL3A1*, and *COL5A2*); some reported virulence genes for TAA (*LOX*, *COL3A1*, and *MMP2*)(Longo et al., 2002; Shen et al., 2015; Pinard et al., 2019; Chen et al., 2022); and potential causative genes (*CTHRC1*, *SERPINH1*, *SPARC*, *THY1*, and *CTSK*) were identified involving in the pathology of TAAs (Figure 3F).

In all, we first identified conserved modules of aortic SMC gene expression within human and mouse SMCs, including contractile SMCs, stressed SMCs, and synthetic SMCs (fibromyocytes and modulated SMCs). Second, our analysis revealed distinct causative genes for TAA and AAA across species in well-conserved SMC subsets (***h*** Fibromyocyte and ***m*** SMC3).

### Macrophages contain common subsets across species

**FIGURE 4:**
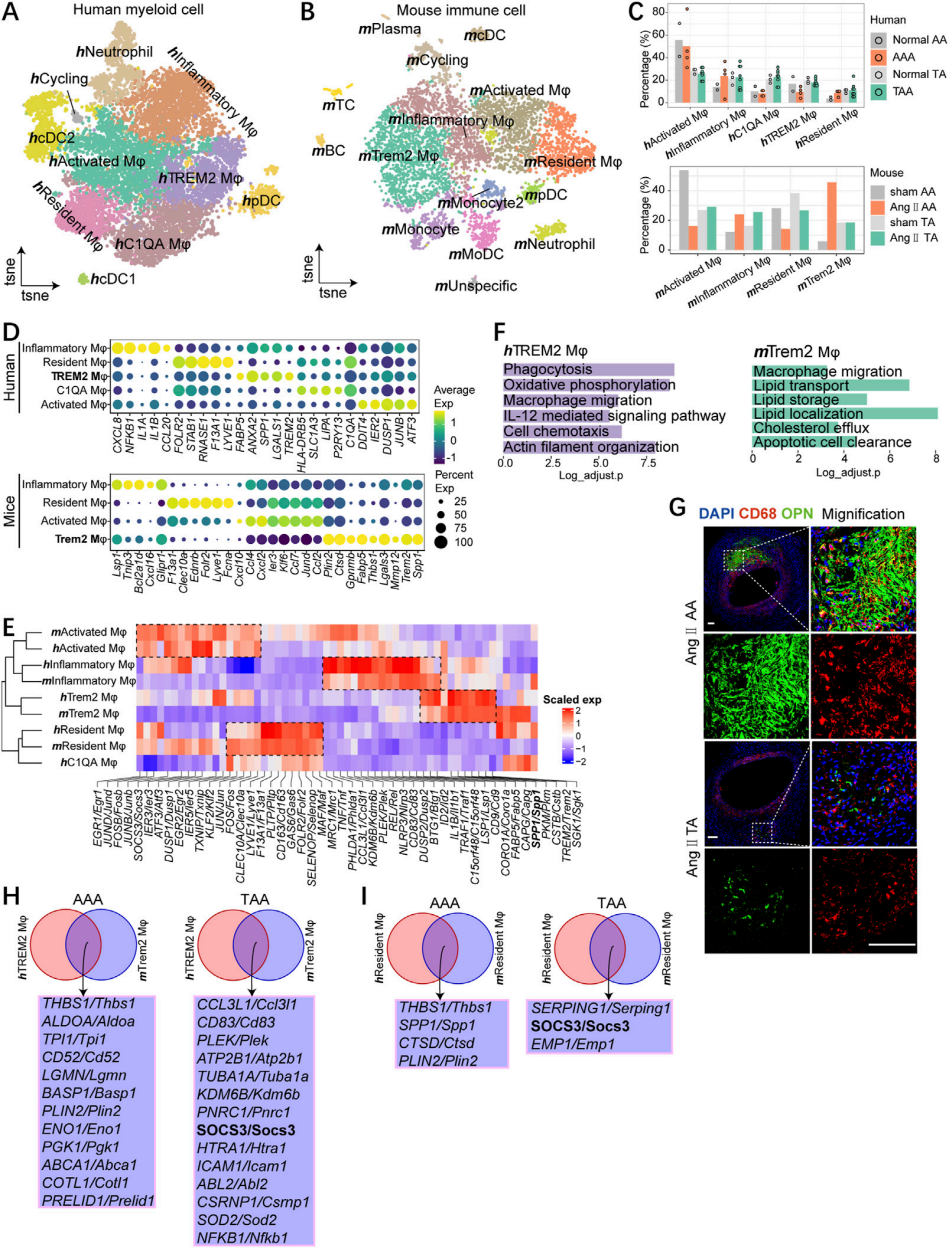
Macrophages contain common subsets across species. **(A)** t-SNE plot, clustering human myeloid cells and showing cell types by color. **(B)**t-SNE plot, clustering mouse immune cells and showing cell types by color. **(C)** Fractions of macrophage subsets among all macrophages of aneurysmal aorta and the corresponding normal aorta of humans (upper) and mice (bottom). **(D)** Dot plot displaying average scaled expression levels (color scaled, column-wise Z scores) of top DEGs (columns) across mouse (bottom) and human (upper) macrophage subsets. The circle size indicates the cell fraction expressing signatures greater than mean; color indicates mean signature expression (yellow, high; blue, low). **(E)** Heatmap showing gene orthologs similarly enriched within mouse and human macrophage subsets. Orthologous mouse and human major cell types established by hierarchical clustering. **(F)** Representative GO terms and pathways enriched in ***h*** TREM2 macrophages (left) and ***m*** Trem2 macrophages (right). **(G)**Immunostaining for CD68 (red), OPN (green), and overlays with DAPI-labeled nuclei (blue) showing ***m*** Trem2 Mφ in the Ang Ⅱ-induced mouse aorta (scale bars: 100 μm). **(H)** Integrated comparative analysis of upregulated DEGs of AAA (left) and TAA (right) between ***h*** TREM2 Mφ and ***m*** Trem2 Mφ. **(I)** Integrated comparative analysis of upregulated DEGs of AAA (left) and TAA (right) between ***h*** Resident Mφ and ***m*** Resident Mφ.

Spectral clustering of patient myeloid cells (without ***h*** mast cell) identified five macrophage subsets, namely, ***h*** Activated Mφ, ***h*** Resident Mφ, ***h*** TREM2 Mφ, ***h*** Inflammatory Mφ, and ***h*** C1QA Mφ, which were defined by unique feature genes and previous publications (Cochain et al., 2018; Weinberger et al., 2020) (Figures 4A, D). The macrophage subsets were all present in each sample, albeit the low cell numbers in the normal group (Supplementary Figure S5A). ***h*** TREM2 Mφ is characterized by higher expression of *SPP1*, *TREM2*, *LGALS1*, *FABP5*, and *ANXA2*, a subpopulation newly identified in atherosclerotic disease (Cochain et al., 2018); ***h*** Resident Mφ expressed genes characteristic of adventitia macrophages (*LYVE*, *1 F13A1*, *RNASE1*, *STAB1*, and FOLR2); ***h*** Inflammatory Mφ was defined by the high expression of chemokines (*CCL20*, *IL1A*, *IL1B*, *CXCL8*, and *NFKB1*); ***h*** Activated Mφ expressed stress response genes including *ATF3*, *JUNB*, *DUSP1*, *IER2*, and *DDIT4*; ***h*** C1QA Mφ is defined by the high expression of complement genes (*C1QA*) (Figure 4D).

In mice, we found four distinct macrophage subsets, which were also annotated by the high expression of characteristic genes (Figures 4B, D; Supplementary Figure S5B). Although the proportion of similar subpopulations varied across species (Figure 4C), these four subpopulations could correspond one-to-one with those found in humans except for ***h*** C1QA Mφ, first by detecting unique markers for each cell type and followed by unsupervised hierarchical clustering using homologous variable genes (Figures 4D, E). ***m*** Resident Mφ mirrored ***h*** Resident Mφ and shared common gene expression patterns (*Lyve1*, *F13a1*, *Pltp*, *Cd163*, *Gas6*, *Folr2*, *Selenop*, *Maf*, and *Mrc1*), whereas ***m*** Trem2 Mφ mirrored ***h*** TREM2 Mφ and featured the high expression of *Spp1* and *Trem2* (Figure 4E)*.* We next analyzed the representative biological functions of each sub-clusters (Supplementary Figure S5C). More importantly, GO term analyses suggested highly specialized functional features of ***m*** Trem2 Mφ, for example, in lipid handling processes, and ***h*** TREM2 Mφ showed enrichment of phagocytosis and cell chemotaxis; both ***m*** Trem2 Mφ and ***h*** TREM2 Mφ enriched for macrophage migration (Figure 4F). We observed accumulative OPN^+^ macrophages (***m*** Trem2 Mφ) in Ang II-induced abdominal aortic aneurysm and several OPN^+^ macrophages (***m*** Trem2 Mφ) in Ang II-induced thoracic aorta (Figure 4G).

To further compare TAA and AAA, we performed comparative analysis and found the TAA group enriched for response to oxidative stress both in ***m*** Trem2 Mφ and ***h*** TREM2 Mφ (Supplementary Figure S5D). In ***h*** Resident Mφ and ***m*** Resident Mφ, the AAA group displayed enrichment for antigen processing and presentation compared with the TAA group (Supplementary Figure S5E). In Trem2 and resident macrophage subsets, we identified different intersected genes between mice and humans. The upregulation of *THBS1* and *PLIN2* both in the Trem2 macrophage and resident macrophage during AAA pathogenesis was observed. Meanwhile, the expression of *SOCS3* upregulated in the TAA group both in Trem2 and resident macrophages, indicating *SOCS3* might be a potential causative regulator gene that participated in the pathological process of TAA.

Generally, four macrophage subpopulations are well-conserved between mice and humans. We identified a subpopulation of the Trem2 macrophage featured biological function on macrophage migration and expression of *SPP1*, *TREM2*, *FABP5*, and *ANXA2* in both organisms during the pathology of aortic aneurysm. Moreover, our analysis indicated THBS1 and *PLIN2* might implicate in AAA, and *SOCS3* might participate in TAA progression.

### High diversity of T cells in human aortic aneurysm

**FIGURE 5:**
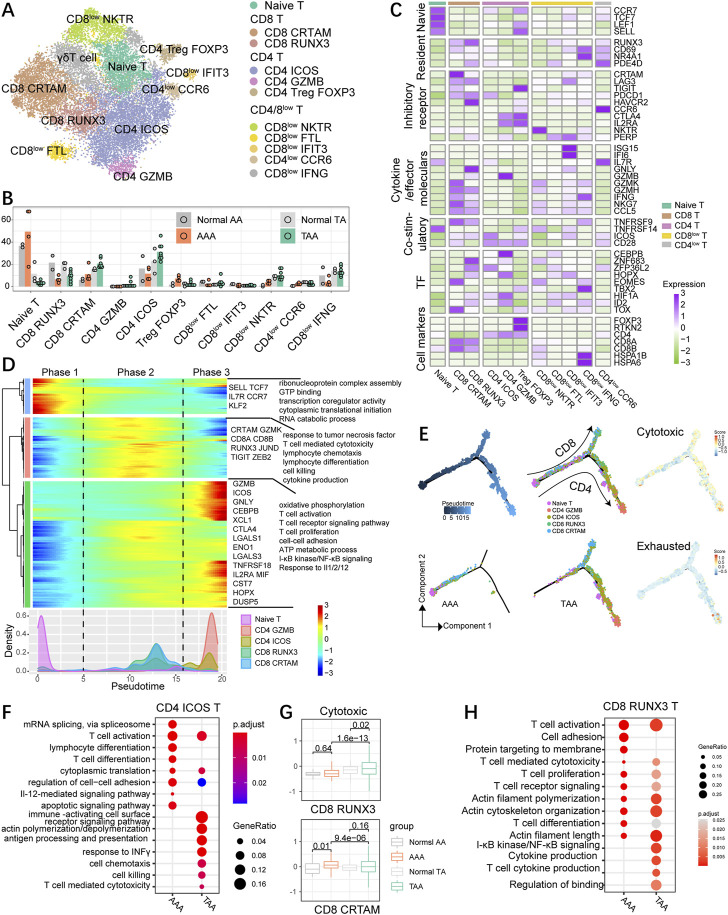
High diversity of T cells in human aortic aneurysm. **(A)** t-SNE plot, clustering human T cells and showing cell types by color. **(B)** Fractions of T-cell subsets among all T cells of the aneurysmal aorta and corresponding normal aorta of humans. **(C)** Heatmap indicating the expression of selected gene sets in T subtypes, including naive cells, resident cells, inhibitory cells, cytokines, co-stimulatory cells, transcriptional factors (TFs), and cell types. **(D)** Heatmap showing the dynamic changes in gene expression along the pseudotime (upper panel). The distribution of the selected T-cell subtypes during the transition (divided into three phases), along with the pseudotime. Subtypes are labeled by colors (bottom panel). **(E)** Pseudotime-ordered analysis of the selected T-cell subsets from AAA and TAA samples, and T-cell subtypes are labeled by colors. 2D pseudotime plot showing the dynamics of cytotoxic or exhausted signals (right panel) in the selected T-cell subsets from human samples. **(F)** Representative GO terms and pathways enriched in upregulated DEGs of CD4 ICOS T cells between TAA and AAA groups. TAA group: TAA vs. Normal TA; AAA group: AAA vs. Normal AA. **(G)** Distribution of normalized expression levels of cytotoxic gene sets of each group in CD8 RUNX3 and CD8 CTRAM subsets. Significance was calculated by the Wilcoxon rank-sum test. **(H)** Representative GO terms and pathways enriched in upregulated DEGs of CD8 RUNX3 T cells between TAA and AAA groups. TAA group: TAA vs. Normal TA; AAA group: AAA vs. Normal AA.

The large and increased proportion of ***h*** T cells revealed its essential role during the aortic aneurysm pathogenesis, and the number of ***m*** T cells is low for further analysis; thus, we performed unsupervised clustering of ***h*** T cells. The re-clustering of ***h*** T cells indicated 11 sub-clusters, containing naïve T cells, two subsets of CD8^+^ T cells (CD8 CRTAM and CD8 RUNX3), three CD4^+^ T-cell subsets (CD4 ICOS, CD4 GZMB, and CD4 Treg FOXP3), four CD8^low^ T-cell subpopulations (CD8^low^ NKTR, CD8^low^ FTL, CD8^low^ IFIT3, and CD8^low^ IFNG), and CD4^low^ CCR6 cells (Figure 5A). Except for the CD4 GAMB subset, other subsets were shared across aneurysm and normal groups, albeit in variable proportions possibly because of the low cell numbers in the normal group (Figure 5B; Supplementary Figures S6A, B).

The diversity of T cells in aneurysm specimens was more abundant than that in non-dilation aortic samples (Figure 5). Naïve T cells with high expression of naive markers, such as *SELL*, *CCR7*, *LEF1*, and *TCF7*, preferentially enriched in abdominal aorta including physiological and pathological conditions. Treg FOXP3 possessed special gene expression properties resembling both Treg and naïve T-cell traits (Figure 5C) with expression of unique Treg hallmarks *FOXP3*, *IL2RA*, *TIGIT*, *TNFRSF14*, and *CTLA4* and blood T-cell-related genes *SELL*, *LEF1*, and *CCR7*, suggesting it as a subset of blood Treg that might exist in aneurysm dissection (Figure 5C). FOXP3^+^ Treg cells were abundant in aortic aneurysms, especially in AAA groups (Figure 5B; Supplementary Figure S6B) and displayed high expression of exhausted genes *CTLA4*, *TIGIT*, *TNFRSF14*, *ICOS*, and *CD28* (Supplementary Figures S6C, D). Among CD4^+^ T cells, CD4 ICOS specifically expressed *ICOS*, *PDCD1*, and *CD28*, suggestive of the state of exhausted CD4 T cells. The fraction of CD4 ICOS was enhanced in TAA but reduced in AAA compared with their corresponding control groups. CD4 GZMB showed low percentage in each group and expressed both cytotoxic (*GZMB*) and exhausted *CTLA4*-related genes. CD4^low^ CCR6 cells might be a cluster of memory-like, tissue-resident T cells on account of the highest expression level of *CD69*, *RUNX3*, and *NR4A1*. In addition, CD8 CTRAM cells highly expressed genes involved in cellular cytotoxicity (*NKG7*, *IFNG*, *GZMK*, *and GZMH*) and exhibited high expression of exhaustion-related markers (*PDCD1*, *TIGHT*, *TNFRSF9*, and *LAG3*) and chemokine genes (*CCL3*, *CCL4*, and *CCL5*) (Figure 5C; Supplementary Figure S6D). CD8 RUNX3 cells displayed enhanced expression of exhaustion-related markers (PDCD1, HAVCR2, *TNFRSF9*, and LAG3) and were typical of the high expression of cytotoxic genes *GNLY*, *NKG7*, and *GZMH* and also displayed a high level of the tissue-resident gene *RUNX3* and a moderate level of tissue-resident genes *NR4A1* and *CD69* (Sun et al., 2021). CD8 CRTAM cells exhibited an escalating trend of proportions both in TAA and AAA than their corresponding control samples, whereas CD8 RUNX3 cells exhibited an opposite trend, namely, decreased proportions in aortic aneurysm compared with the normal group. We also identified that the gene expression patterns of CD8^low^ FTL cells was similar to the identified CD8 CX3CR1 cells (Zheng et al., 2017). CD8^low^ NKTR and IFIT3 cells showed the intermediate expression level of cytotoxic-related genes *GZMH*, *GZMK*, *NKG7*, and *IFNG* and also expressed low levels of the checkpoint genes (*GTLA4*, *HAVCR2*, and *TIGIT*), indicating that they might be precursors of cytotoxic T cells. CD8^low^ IFNG resembled tissue-resident CD8^+^ effector cells due to high abundance of effector cell hallmark *IFNG* and resident genes *CD69* and *NR4A1*, and the mediate expression of the cytotoxic genes. CD8^low^ IFNG also highly expressed genes linked to stress response, such as *FOS*, *JUN,* and genes encoding heat shock proteins (HSPs), including *HSPA1B*, *HSPA1A*, and *HSP90AA1.*

We next explored the dynamic immune state and cell transitions in naïve, CD4^high^ (except for blood Treg), and CD8 ^high^ T cells by using Monocle to infer cell state pseudotime trajectories. We excluded CD4 Treg on account of the high expression of blood T-cell markers. This analysis indicated that naïve T cells existed at the start of the pseudotime trajectory path (phase 1), typical of upregulated expression of *SELL*, *CCR7*, *TCF7*, *IL7R*, and *KLF2*, whereas the CD4 GZMB cells and partial CD4 ICOS cells at a terminal state (phase 3); CD8 CTRAM and RUNX3 cells were predominately at the intermediate pseudotime (phase 2) and distributed in another branch (Figures 5D, E), indicating that CD4 and CD8 T cells exhibit distinct differentiating trajectory. Pathway analysis demonstrated that signaling pathways associated with T-cell-mediated cytotoxicity and killing, lymphocyte differentiation, cytokine and chemotaxis production, and response to tumor necrosis factor were enriched in phase 2. Phase 3 cells were characterized by high expression of transcriptional factors *CEBPB*, *HOPX*, and stress response genes *DUSP5* and *ENO1*, and enriched for biological functions on T-cell activation, proliferation, and migration (cell–cell adhesion), T-cell receptor signaling pathway, and oxidative phosphorylation (Figure 5D). Both CD4 and CD8 T cells expressed the upregulated cytotoxic signature during the transition process, and CD4 cells expressed little higher exhausted gene set scores than CD8 cells (Figure 5E). To further characterize the transition state linked to CD4 and CD8 T cells in aneurysm groups, we inferred the movement trajectories of CD4 and CD8 T cells in the TAA and AAA groups, respectively. Significantly, early-stage CD4/CD8^+^ T cells were primarily present at AAA groups, with few cells found at the terminal point of the state transition path, while T cells in TAA groups were mainly distributed at the ends of both branches (Figure 5E).

To further compare TAA and AAA in different CD4/CD8 T-cell subsets, we conducted and compared GO analysis in the CD4 ICOS subgroup and uncovered common biological function on T-cell activation and migration supported by enrichment in cell–cell adhesion, cytoplasmic translation, and actin polymerization/depolymerization; the TAA group uniquely enriched in response to INFγ, T cell-mediated cytotoxicity, chemotaxis, and cell killing (Figure 5F). Among CD8 T cells, CD8 CTRAM in the AAA group exhibited abundance in cytotoxic genes compared with the normal AA group, while TAA significantly exacerbated cytotoxicity in CD8 RUNX3 T cells (Figure 5G). Consistently, CD8 RUNX3 T cells in the TAA group displayed upregulation of cytokine production and NF-κB signaling (Figure 5H).

We conclude that CD4^+^ and CD8^+^ cells in thoracic and abdominal aortic aneurysm samples showed distinct transition trajectories and subsets and displayed distinct inflammatory and gene transcriptional situations, indicating that targeted treatment strategies should be taken into consideration for the therapy of TAA and AAA.

### MIF and SPP1 signaling pathways commonly altered among different species

**FIGURE 6:**
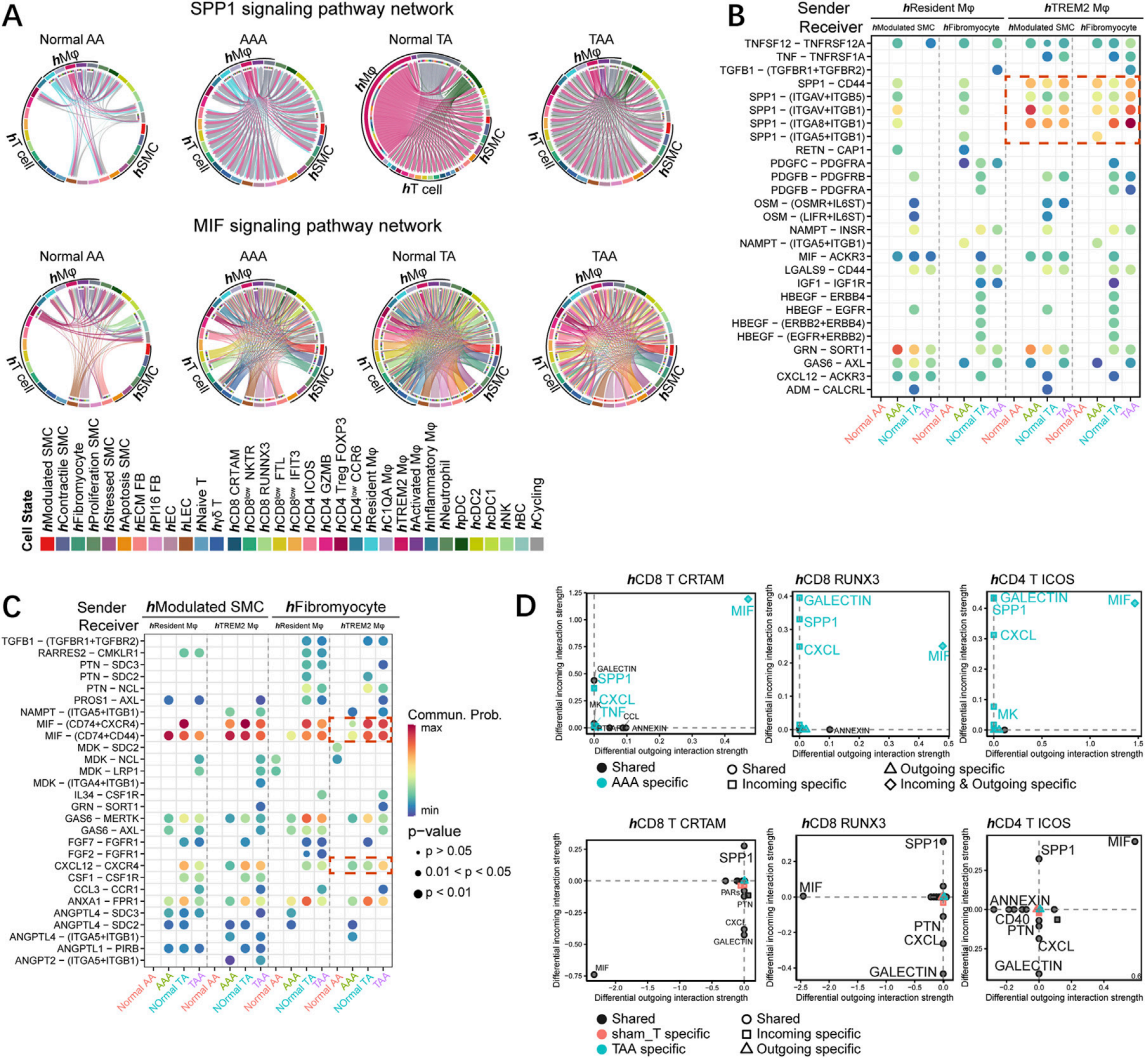
MIF and SPP1 signaling pathways commonly altered among different species. **(A)** Chord diagram of SPP1 (upper) and MIF (bottom) signaling networks in human samples (normal AA, AAA, normal TA, and TAA). **(B)** Comparison of the significant ligand–receptor pairs between normal AA, AAA, normal TA, and TAA, which contribute to the signaling from ***h*** Resident Mφ and ***h*** TREM2 Mφ to ***h*** Modulated SMC and ***h*** Fibromyocte. The dot color reflects communication probabilities, and the dot size represents computed *p*-values. Empty space means the communication probability is zero. *p*-values are computed from the one-sided permutation test. **(C)** Comparison of the significant ligand–receptor pairs between normal AA, AAA, normal TA and TAA, which contribute to the signaling from ***h*** Modulated SMC and ***h*** Fibromyocte to ***h*** Resident Mφ and hTREM2 Mφ. **(D)** Signaling changes of T-cell subpopulations (***h*** CD8 CTRAM, ***h*** CD8 RUNX3, and ***h*** CD4 ICOS) in AAA (upper) and TAA (bottom) compared to the respective control group.

MIF and SPP1 signaling involved both in TAA and AAA progress among different species based on cell–cell communication. SPP1 signaling mainly sent from macrophages and became much more abundant in the aortic aneurysm group in both organisms, although ***h*** TREM2 Mφ in the normal group showed strong SPP1 signaling (Figure 6A; Supplementary Figure S7A). MIF signaling originated from ***h*** SMC and ***h*** T cell appeared in the AAA group compared with the normal AA group (Figure 6A, bottom). Meanwhile, MIF signaling was produced by different cell populations and received by different cell populations in the human TAA group and Ang II-induced aortic aneurysm (Figure 6A; Supplementary Figure S7A). Among the ligand–receptor pairs between the macrophage subset (***h*** Resident Mφ and ***h*** TREM2 Mφ) and SMC subsets (***h*** Modulated SMC and ***h*** Fibromyocyte), consistently, several ligand–receptor pairs (SPP1-CD44, SPP1-ITGAV+ITGB5, SPP1-ITGA8+ITGB1, and SPP1-ITGA5+ITGB1) of SPP1 signaling sent from ***h*** TREM2 Mφ, received by SMC subsets, were obviously upregulated both in TAA and AAA compared with their respective normal groups (Figure 6B). On the other hand, MIF- (CD74+CXCR4) and MIF- (CD74^+^CD44) ligand–receptor pairs were significantly enriched in signaling sent from SMC subsets and received by macrophage subsets in the AAA group compared with the normal AA group, while the enrichment of these two ligand–receptor pairs in thoracic aortic aneurysm only presented in ***h*** Fibromyocte and ***h*** TREM2 Mφ (Figure 6C). In line with previous research (Michineau et al., 2014), the CXCL12-CXCR4 ligand–receptor pair was abundant among T-cell subsets, macrophage subsets, and SMC subsets (Figure 6C; Supplementary Figures S7C, D). In mice, we also observed the upregulation of MIF secreted by ***m*** SMC3 in TAA, whereas the difference with human samples was that the elevation of the ligand SPP1 was observed in ***m*** Resident Mφ, ***m*** SMC3, and ***m*** SMC4 but not ***m*** Trem2 Mφ after Ang II stimulation across segments (Supplementary Figure S7B).

We then focused on the T-cell subset and found the specific increase of both incoming and outcoming MIF in CD8 CRTAM, CD8 RUNX3, and CD4 ICOS T cells in the AAA group (Figure 6D). Incoming MIF signaling received by T-cell subsets mainly originated from ***h*** Modulated SMC and ***h*** Fibromyocte, and MIF- (CD74+CXCR4) and MIF- (CD74^+^CD44) ligand–receptor pairs were more abundant across T-cell subsets in AAA than those in normal AA (Supplementary Figure S7C). Moreover, SPP1 signaling received by these three T-cell subsets was specifically enhanced in the AAA group; further analysis indicated the SPP1 secreted by ***h*** TREM2 Mφ significantly potentiated. SPP1-CD44 ligand–receptor pairs participated in the interaction of ***h*** TREM2 Mφ and T-cell subsets (Figure 6D; Supplementary Figure S7D). Different from AAA, SPP1 and MIF signaling also enriched in the normal TA group; thus, both TAA and normal TA groups shared SPP1 and MIF incoming and outcoming in ***h*** CD8 CRTAM, ***h*** CD8 RUNX3, and ***h*** CD4 ICOS T-cell subsets (Figure 6D, bottom). However, MIF- (CD74+CXCR4) and MIF- (CD74^+^CD44) ligand–receptor pairs sent from ***h*** Modulated SMC and ***h*** Fibromyocte, received by T-cell subsets showed increased expression in TAA compared with normal TA (Supplementary Figure S7C). SPP1-CD44 ligand–receptor pairs, originated from ***h*** TREM2 Mφ, received by T-cell subsets also displayed enhanced expression in TAA compared with normal TA (Supplementary Figure S7D).

In all, SPP1 and MIF signaling between macrophage and SMC subsets might exert an essential role during aneurysm progress among different species. Furthermore, although the alteration of SPP1 and MIF signaling pathways in T-cell subsets was distinct between TAA and AAA, the common increase of ligand–receptor pairs containing MIF-(CD74+CXCR4), MIF- (CD74^+^CD44), and SPP1-CD44 was observed across segments.

## Discussion

In this study, we compared aneurysmal aortic cell cluster structures between humans and mice by scRNA-seq, without biasing the notions of pre-defined marker genes and cell states. In doing so, our group depicted the aortic aneurysm transcriptional profiles in each organism, displaying both known and unappreciated aortic aneurysm disease-associated biological functions and marker genes. In discussing this work, we begin with our findings regarding aortic aneurysm homogeneity and dissimilarity across species and segments, conservation of SMC and macrophage subsets between organisms, and distinct marker genes for TAA and AAA. Both SMCs and macrophages contained conserved subpopulations across species. We additionally uncovered different biological functions and distinct causative genes for TAA and AAA. Moreover, high diversity of T cells in human aortic aneurysms was described. MIF and SPP1 signaling networks participated in aortic aneurysm in both organisms. Thus, we provide basic information for understanding the mechanisms of cellular compositions and gene profiles for both TAA and AAA.

SMCs are the main cell components of the aorta wall, and their loss by necroptosis or apoptosis is a common decisive feature of both TAA and AAA (Quintana and Taylor, 2019). Consistently, our data demonstrated that the fraction of SMCs was remarkably reduced both in TAA and AAA among different species. Similar to AAA, TAA is characterized by ECM abnormalities that disrupt the structural integrity of the aorta. Accumulative studies have shown that genetic variants in proteins (COL3A1, FIBRILLIN-1, and so on) directly affect the mechanical properties of the aorta, ultimately leading to TAA (Malfait, 2018), reflecting that adventitial fibroblasts, as the main producer of ECM, might be related to the pathogenesis of aneurysm. In Ang II-induced mouse aortic aneurysm, we observed significant fibroblast expansion in pathological conditions. However, the proportion of fibroblasts was reduced in human AAA, which might be caused by the low number of AAA samples and the small cell counts. As for immune cells, a hallmark of abdominal aortic aneurysm formation is a strong inflammatory response, including almost all classical inflammatory cell components, as well as resident inflammation in the arterial wall, e.g., neutrophils, macrophages, and T-cell infiltration (Eliason et al., 2005; Rateri et al., 2011; Ait-Oufella et al., 2013). T cells and macrophages existed in the media of human aneurysmal thoracic aorta, although there is less data supporting the function for inflammatory cells in TAA (He et al., 2008). Different from human samples, the number of T cells in mouse samples is very low, although the disease group is more numerous than the normal group. Our findings, to some extent, illustrate the similarity between thoracic and abdominal aortic aneurysms in terms of inflammatory infiltration, as well as the inadequacy of the Ang II-induced aneurysm model in studying T cells.

Although the single-cell map of SMCs and macrophages in aortic aneurysm has been reported (Zilionis et al., 2019; Davis et al., 2021; Liu et al., 2022; Yu et al., 2022), we reveal an innovative conservation of gene expression profiles between mice and humans. First, we see coherence of SMC subpopulations. The ***h*** Fibromyocyte and ***m*** SMC3 highly express innovative marker genes well conserved across species, including *CTHRC1*, *COL8A1*, and *LGALS3*, which involves in phenotypic switching into synthetic SMC. The high similarities of gene expression profiles between ***h*** Contractile SMC and ***m*** SMC1, ***h*** Stressed and ***m*** SMC2, and ***h*** Modulated SMC and ***m*** SMC4 are observed. Second, macrophages show striking coherence between organisms. Unsupervised clustering identifies five macrophage subsets ***h*** Activated Mφ, ***h*** Resident Mφ, ***h*** TREM2 Mφ, ***h*** Inflammatory Mφ, and ***h*** C1QA Mφ in humans and four subsets in mice, and we, indeed, observe four subpopulations showing a one-to-one correspondence between mice and humans. Both ***m*** Trem2 Mφ and ***h*** TREM2 Mφ are characterized by high expression of *SPP1* and *TREM2*, respectively, and enrich for macrophage migration. Furthermore, in TAA and AAA, we identify distinct marker genes as potential regulators participating in the pathological process in both organisms. For example, both in ***h*** Fibromyocyte and ***m*** SMC3, we identify potential causative genes for AAA (*CXCL12*, *MFAP5*, and *EMP1*), among which the blockade of CXCL12/CXCR4 protects against AAA formation (Michineau et al., 2014), and distinct pathogenic genes for TAA [collagen genes (*COL1A1*, *COL1A2*, *COL3A1*, and *COL5A2*), some reported virulence genes for TAA (*LOX*, *COL3A1*, and *MMP2*) (Longo et al., 2002; Shen et al., 2015; Pinard et al., 2019; Chen et al., 2022), and other unreported genes (*CTHRC1*, *SERPINH1*, *SPARC*, *THY1*, and *CTSK*)]. Among the genes we enriched, the presence of these proven disease-causing genes to some extent supported the validity of our analysis, and the role of these unreported genes deserved further investigation. Generally, the emergence of the congruent expression patterns in two different species encourages us to establish a treatment approach for the aneurysm biological relation between the laboratory and clinics.

A limited number of reports suggest T-cell depletion attenuates AAA formation (Xiong et al., 2004). Regulatory T cells protect against AAA formation by secreting inflammatory cytokines IL-10 and TGF-β (Wang et al., 2010; Yodoi et al., 2015; Zhou et al., 2015). Conflicting reports suggest that CD4^+^ T-cell-derived IFN-γ exerts the function on inducing AAA and preventing from AAA formation, and the exact function of CD8^+^ T cell in AAA remains elusive (Li et al., 2018). Patients treated with doxycycline for 2 weeks prior to open AAA surgery experienced a 95% decrease in cytotoxic T-cell counts on AAA biopsies compared to the control group (Lindeman et al., 2009). Furthermore, little is known about the function of T cells on TAA. Here, we defined T-cell subsets by the unique expression genes. CD4^+^ T cells and CD8^+^ T cells showed distinct transition trajectories and subsets and displayed different immune and transcriptional states in thoracic and abdominal aortic aneurysm samples. CD8 CTRAM and CD8 RUNX3 might have exerted opposite functions during the process of thoracic and abdominal aortic aneurysms because of the opposite trend on fraction alterations. In addition, except for shared biological functions, T-cell subsets in TAA and AAA exhibited distinct functions, for example, CD4 ICOS in the TAA group uniquely enriched for cellular response to INF-γ compared with the AAA group. Therefore, the findings could shed light on using recognized sub-populations as potential innovative therapeutic targets and investigating them in mice.

We note three limitations to this study. First of all, profiling more AAA and normal AA patients could be more convincing. We could only collect two normal AA and four AAA scRNA-seq datasets. The heterogeneity of human samples and the small number of cells may lead to the deviation of the results of cell composition analysis. Second, unbiased comparison analysis of T-cell subsets between mice and humans are not performed due to the very small absolute number of mouse T cells. Third, in comparison with humans, one mouse model system of Ang II induction is used, and other mouse aneurysm models were not compared. Although there is a considerable degree of commonality between species, the utilization of animal models to study the pathological mechanisms of human aneurysm disease is still inadequate. Therefore, the unbiased comparison method developed here between different species can be applied to other mouse models, which helps select and/or prove animal models associated with human aortic aneurysm pathogenesis.

Despite these technical challenges and limitations, our study yielded some innovative and interesting findings. We revealed similarities and differences of changes in the components of human and mouse cell types, and our unbiased comparison between mice and humans identified well-conserved subpopulations of SMCs and macrophages. Furthermore, the results of our comparative analyses suggested different biological functions and distinct marker genes for TAA and AAA. These discoveries expand our understanding of thoracic and abdominal aortic aneurysm pathogenesis in both organisms and may contribute to the development of novel treatments.

## Data Availability

The datasets presented in this study can be found in online repositories. The names of the repository/repositories and accession number(s) can be found in the article/Supplementary Material. To review GEO accession GSE221789: Go to https://www.ncbi.nlm.nih.gov/geo/query/acc.cgi?acc=GSE221789 Enter token ihgbyaaynjgjtmp into the box.
