# Supplementary material for: Comparative analysis of thoracic and abdominal aortic aneurysms across the segment and species at the single-cell level
Source: Front Pharmacol. 2023 Jan 10;13:1095757. doi: 10.3389/fphar.2022.1095757 (PMC9871934; doi:10.3389/fphar.2022.1095757)
Supplement: Supplementary file 1 [file Table2.docx]

**Comparative analysis of thoracic and abdominal aortic aneurysm across segment and species at single-cell level**

Hong Wu^1^, Cheng Xie^2^, Ruilin Wang^1^, Jun Cheng^2^, Qingbo Xu^1^ and Haige Zhao^3^

^1^Department of Cardiology, the First Affiliated Hospital, Zhejiang University School of Medicine, Hangzhou, China;

^2^Key Laboratory of Medical Electrophysiology of Ministry of Education and Medical Electrophysiological Key Laboratory of Sichuan Province, Collaborative Innovation Center for Prevention and Treatment of Cardiovascular Disease, Institute of Cardiovascular Research, Public Center of Experimental Technology, Southwest Medical University, Luzhou, China;

^3^Department of Cardiothoracic Surgery, the First Affiliated Hospital, Zhejiang University School of Medicine, Hangzhou, China

**Running title:** Thoracic and abdominal aortic aneurysm

**Word Count**: 7,863 words.

**Addresses for Correspondence:**

Qingbo Xu, MD, PhD

Department of Cardiology, the First Affiliated Hospital, Zhejiang University Medical School, 79 Qingchun Road, Hangzhou 310003, Hangzhou, China, Email: qingbo_xu@zju.edu.cn, Tel: +86 571-87236500, Fax: +86 571 4008306430

Or Haige Zhao, MD, Department of Cardiothoracic Surgery, the First Affiliated Hospital, Zhejiang University School of Medicine, Hangzhou, China, Email: [haigezhao@zju.edu.cn](mailto:haigezhao@zju.edu.cn)

**Supplementary Figures**


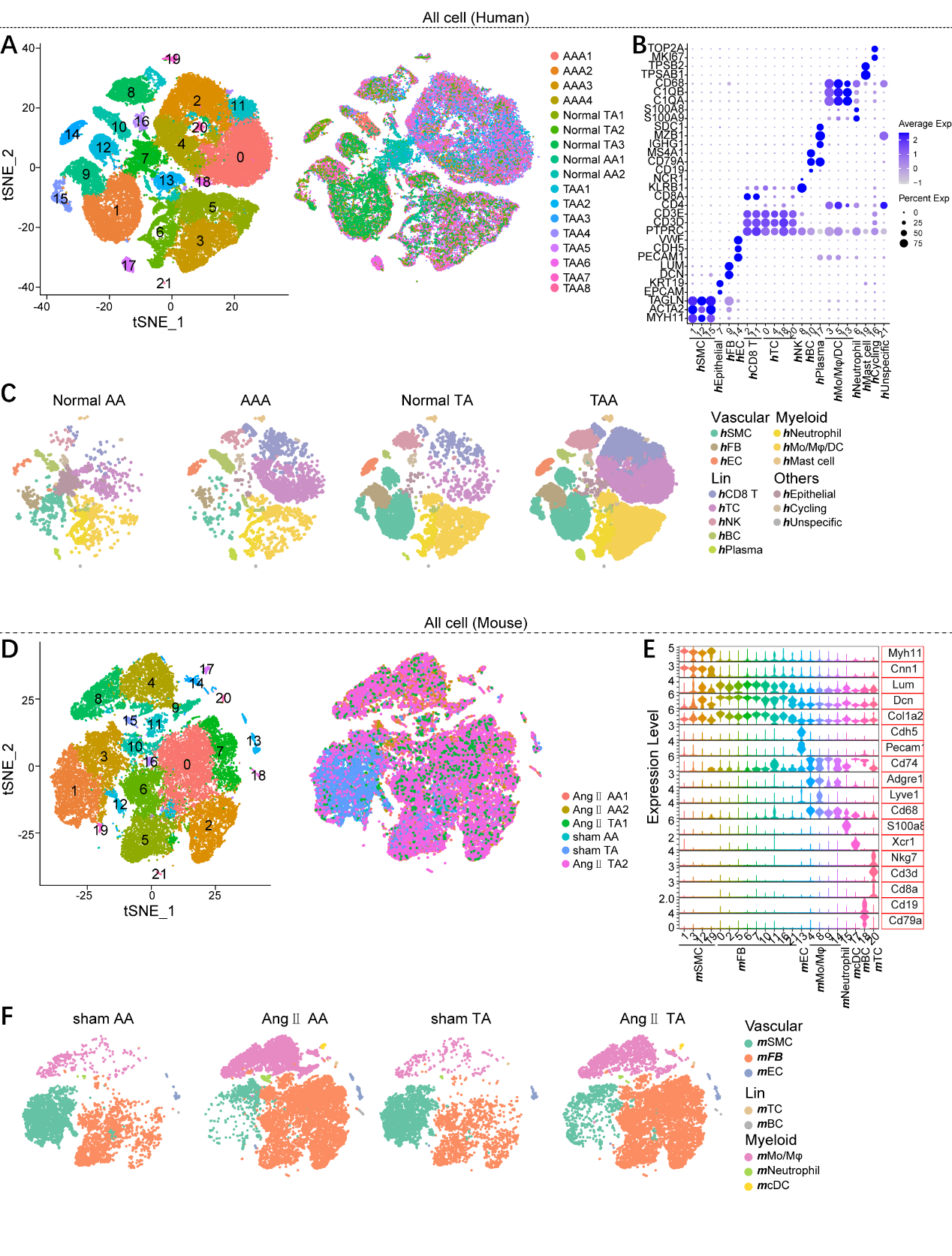


**Figure S1: scRNA-seq Profiling Maps Cell Gene Expression in Human and Mouse Aortic Aneurysm.**

1. The t-SNE plot, clustering all aorta cells from human TAA patients (38,681 cells, n = 8 patients) and normal thoracic aorta (8,297 cells, n = 3 patients), AAA patients (7,257 cells, n = 4 patients) and normal abdominal aorta (4,815 cells, n = 2) (left panel), and showing cell origins by color, patient origin (right panel).
2. Dot-plot: annotating clusters by lineage signatures. Circle size indicates cell fraction expressing signature greater than mean; color indicates mean signature expression (blue, high; grey, low).
3. Split view of major cell types from human aorta by disease condition.
4. The t-SNE plot, clustering all aorta cells from mouse Ang Ⅱ-induced thoracic aorta (11,438 cells, n = 2 samples) and sham thoracic aorta (5,187 cells), Ang Ⅱ-induced abdominal aneurysm (13,102 cells, n = 2 samples) and sham abdominal aorta (5,804 cells) (left panel), and showing cell origins by color, patient origins (right panel).
5. Violin plot annotating clusters of mice aorta cells by lineage signatures.
6. Split view of major cell types from mouse aorta.


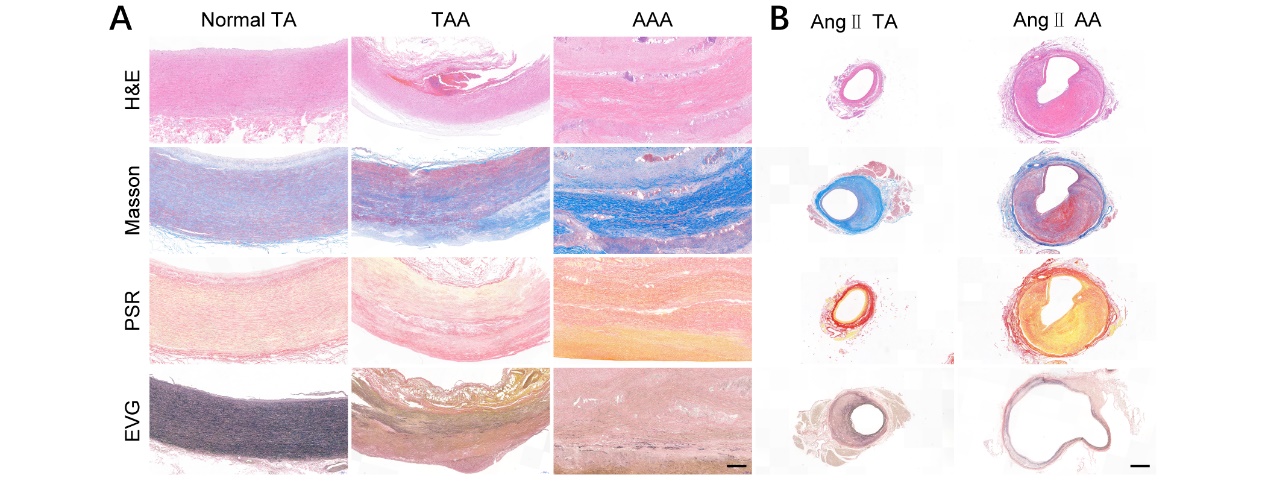


**Figure S2: Histological Characteristics of Aortic Aneurysm of Mouse and Human.**

1. H&E stain, masson trichrome stain, picrosirius red stain and verhoeff stain of aortic wall from aortic aneurysm patients (n =4, TAA group; n=1, AAA group) and non-dilated aorta (Scale bar: 500 μm).
2. H&E stain, masson trichrome stain, picrosirius red stain and verhoeff stain of aortic wall from Ang Ⅱ-induced mouse aortic aneurysm and sham group (Scale bar: 500 μm).


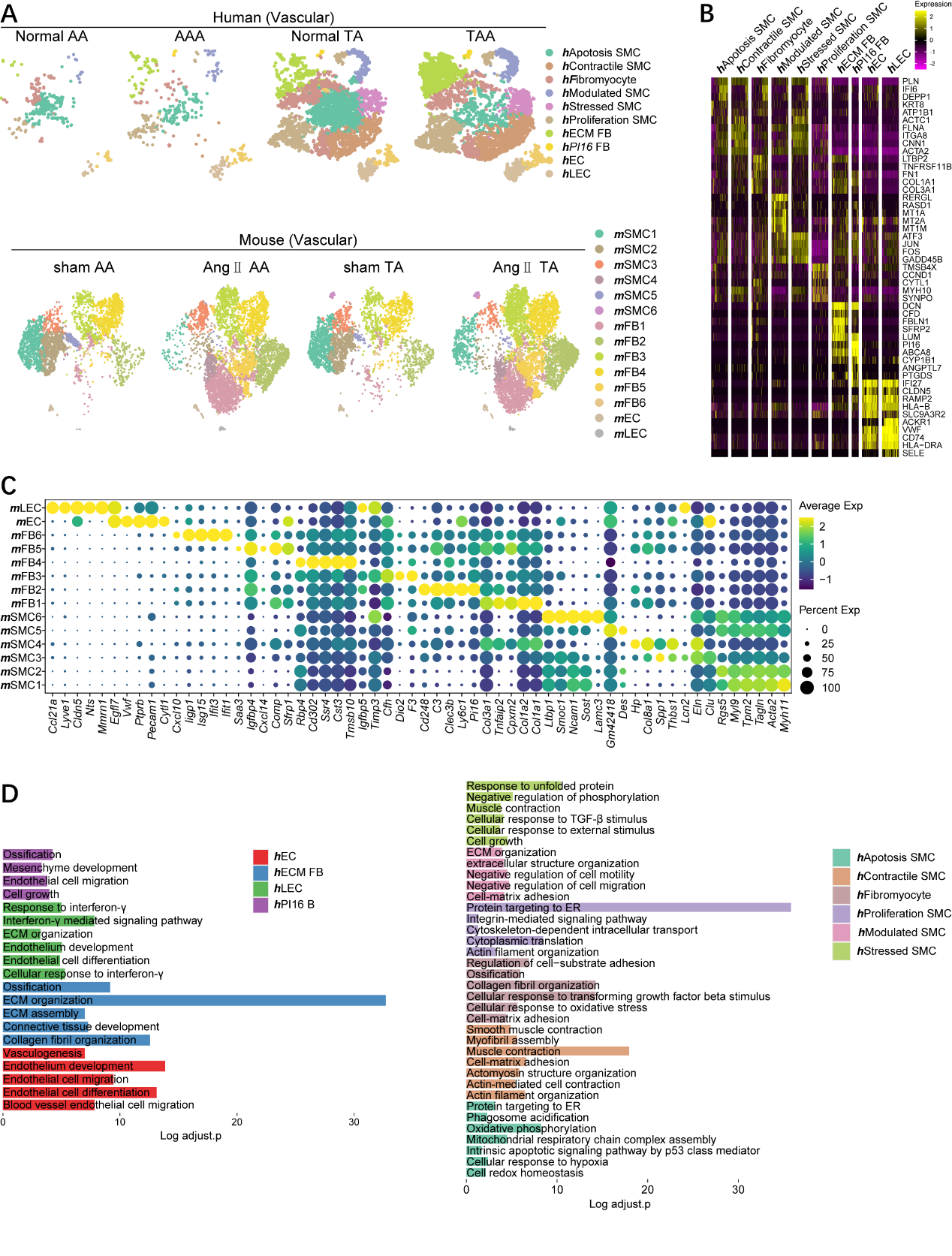


**Figure S3: Analysis of Subsets of Vascular Cells from Mouse and Human.**

1. Split view of subpopulations of vascular wall cells from human (upper) and mouse (bottom) aorta.
2. Heatmap showing the expression of top 5 marker genes in each subsets of human vascular wall cells.
3. Dotplot displaying average scaled expression levels (color-scaled, column-wise Z scores) of top DEGs (columns) across subsets of mouse vascular wall cells. Circle size indicates cell fraction expressing signature greater than mean; color indicates mean signature expression (yellow, high; blue, low).
4. Bar plot showing specific gene ontology biological process enriched in sub-clusters of human fibroblasts, ECs, LECs (left) and SMCs (right).


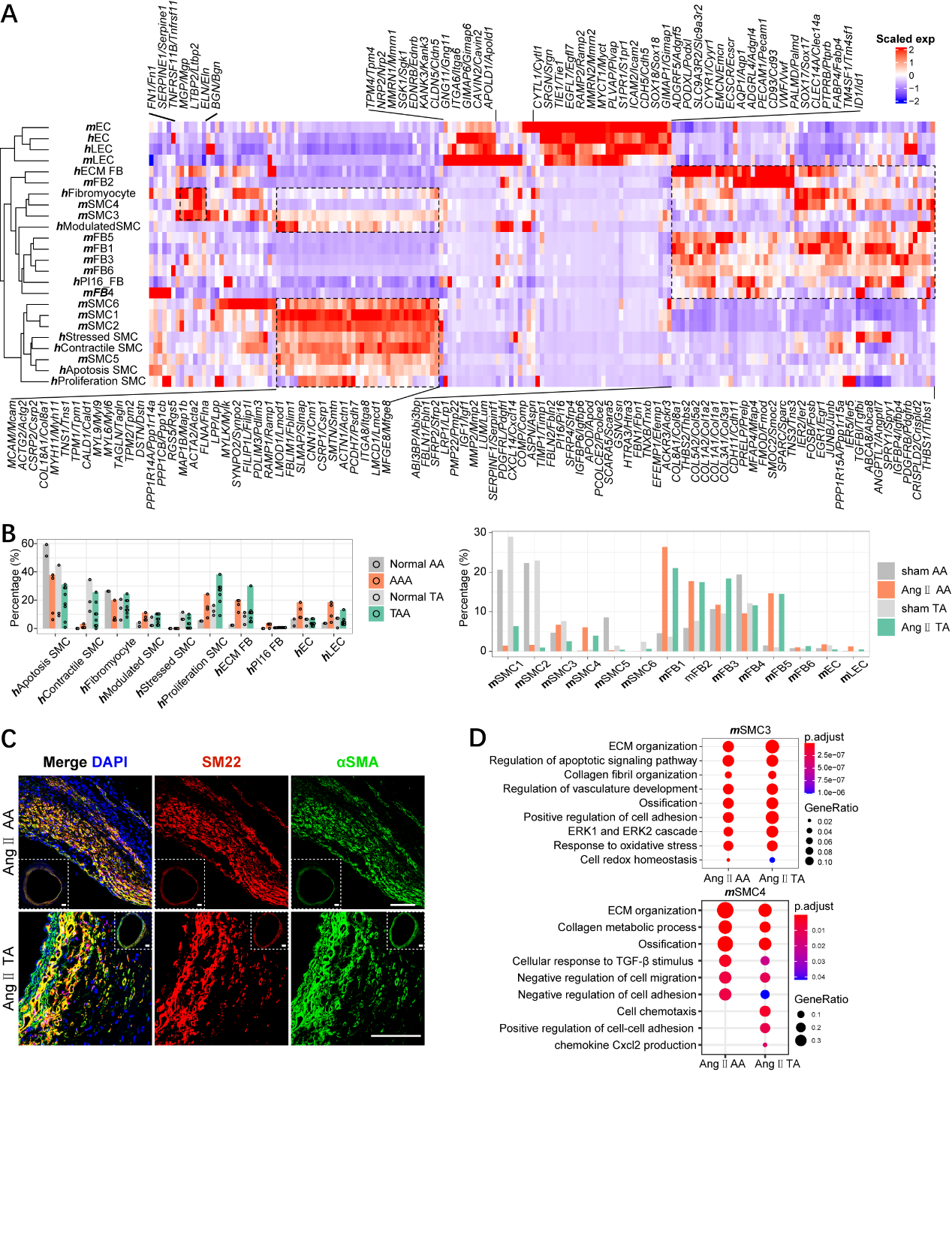
**Figure S4: Unbiased Comparison between Mouse and Human Vascular Wall Cells.**

1. Comparison of mouse and human vascular cell subsets. Orthologous mouse and human vascular cell subsets established by hierarchal clustering. Heatmap showing genes similarly enriched within mouse and human vascular wall cell subsets. The gene set and scaled exp defined as in Figure 1F.
2. Fractions of subsets of vascular wall cells in each dataset of aneurysmal aorta and corresponding normal aorta of human (left) and mouse (mouse).
3. Representative immunofluorescence images: SM22 (red), αSMA (green) and DAPI (blue), scale bar 100 μm.
4. Representative GO terms and pathways enriched in up-regulated DEGs of ***m***SMC3 (upper) and ***m***SMC4 (bottom) between TAA and AAA groups.


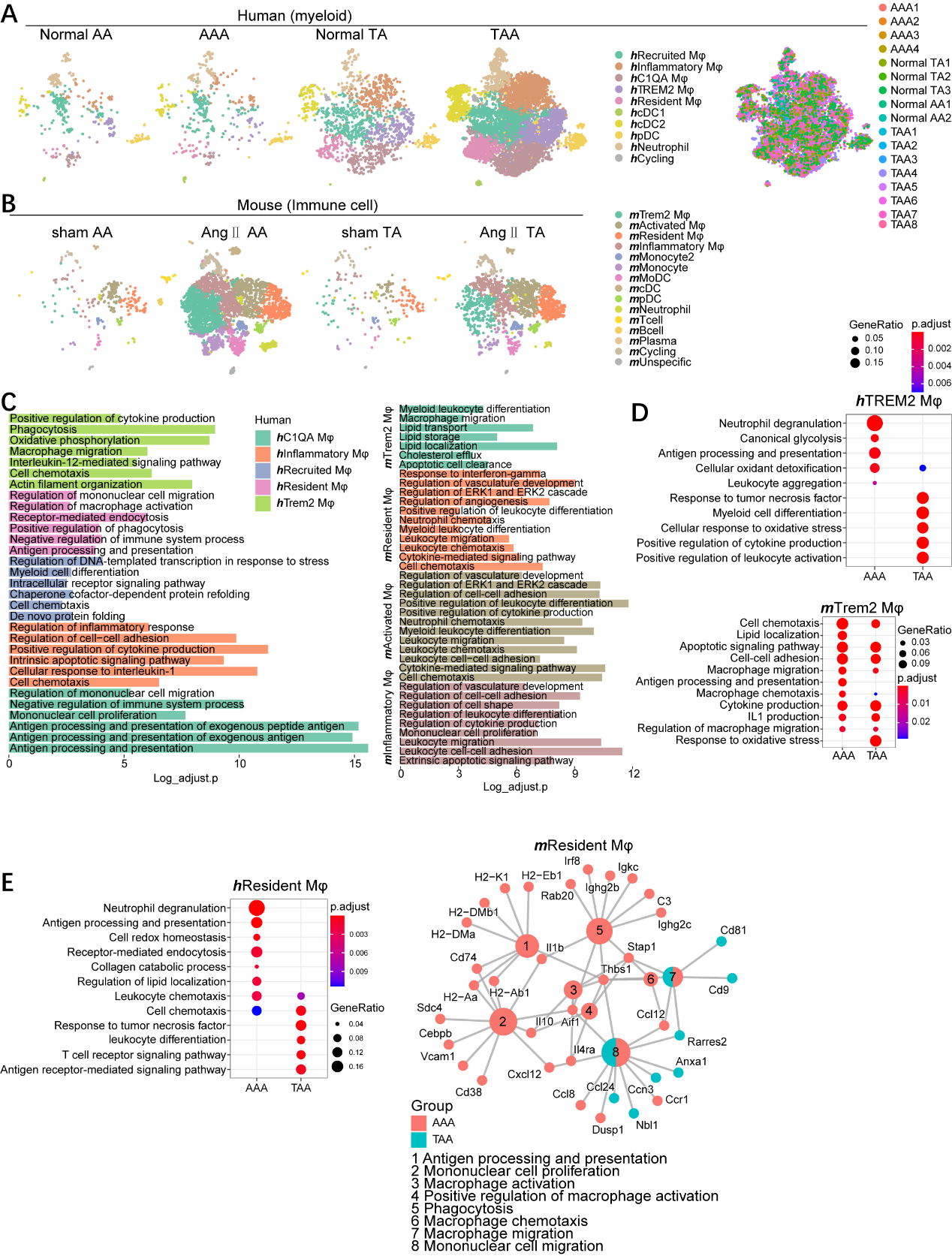


**Figure S5: Macrophages Contain Common Subsets across Species.**

1. Split view of subpopulations of macrophages from human aorta (left); the t-SNE plot, clustering human macrophages and showing cell origins by color.
2. Split view of subpopulations of macrophages from mouse aorta.
3. Bar plot showing specific gene ontology biological process enriched in sub-clusters of human macrophages (left) and mouse macrophages (right).
4. Representative GO terms and pathways enriched in up-regulated DEGs of ***h***TREM2 Mφ (upper) and ***m***Trem2 Mφ (bottom) between TAA and AAA groups. TAA group: TAA vs Normal TA; AAA group: AAA vs Normal AA.
5. Representative GO terms and pathways enriched in up-regulated DEGs of ***h***Resident Mφ (left) and ***m***Resident Mφ (right, cnetplot) between TAA and AAA groups. TAA group: TAA vs Normal TA; AAA group: AAA vs Normal AA.


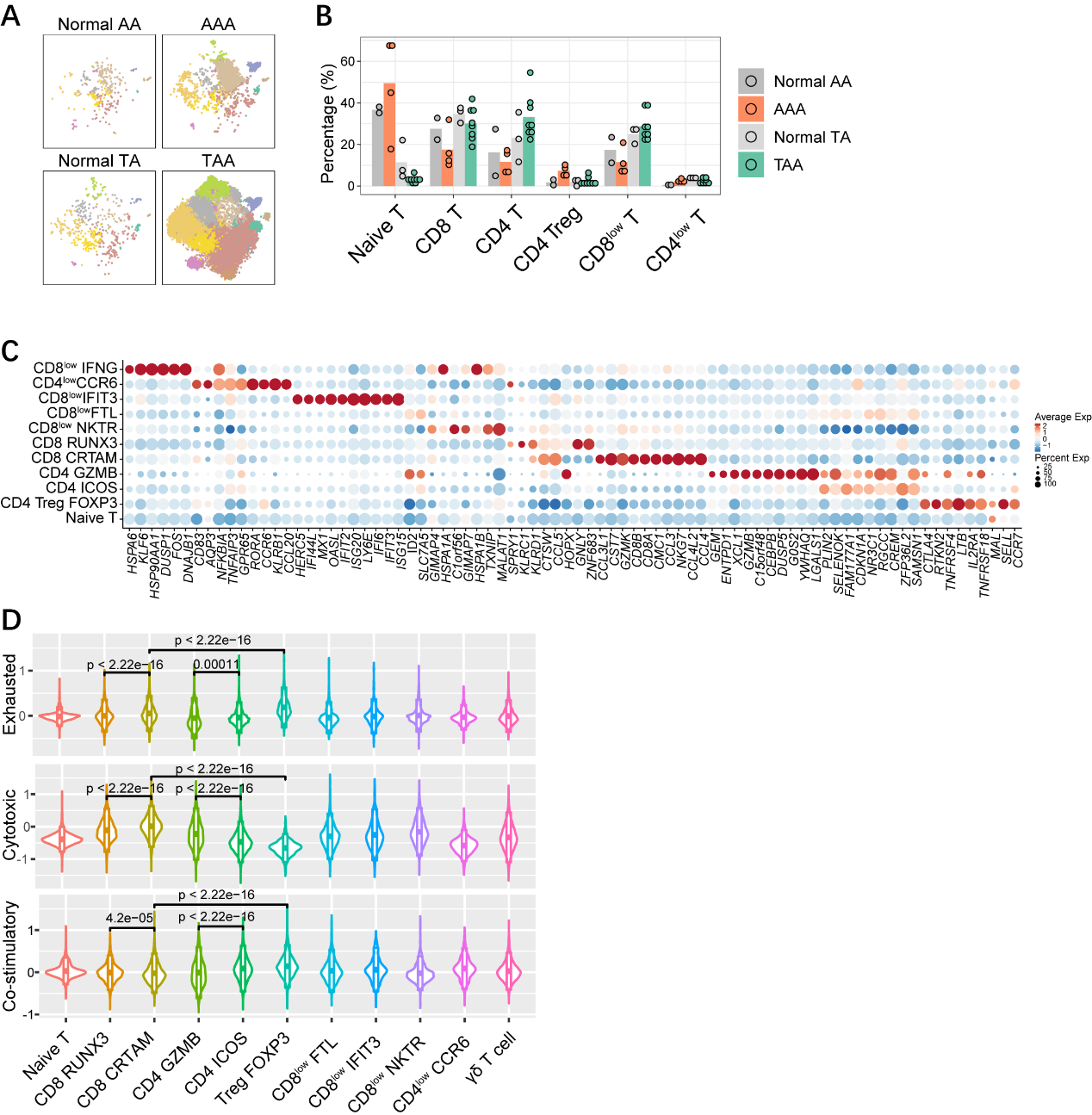


**Figure S6: High Diversity of T cells in Human Aortic Aneurysm.**

1. Split view of subgroups of T cells in each group.
2. Fractions of naïve T cells, CD4^+^ T cells, CD8^+^ T cells, CD4^low^ T cells, CD8 ^low^ T cells among all T cells of aneurysmal aorta and corresponding normal aorta of human.
3. Dotplot displaying average scaled expression levels (color-scaled, column-wise Z scores) of top DEGs (columns) across T cell subsets. Circle size indicates cell fraction expressing signature greater than mean; color indicates mean signature expression.
4. Distribution of normalized expression levels of cytotoxic, exhausted and co-stimulatory gene sets in each subset of T cells. The p value indicated in the plot was calculated by a paired Wilcoxon test.


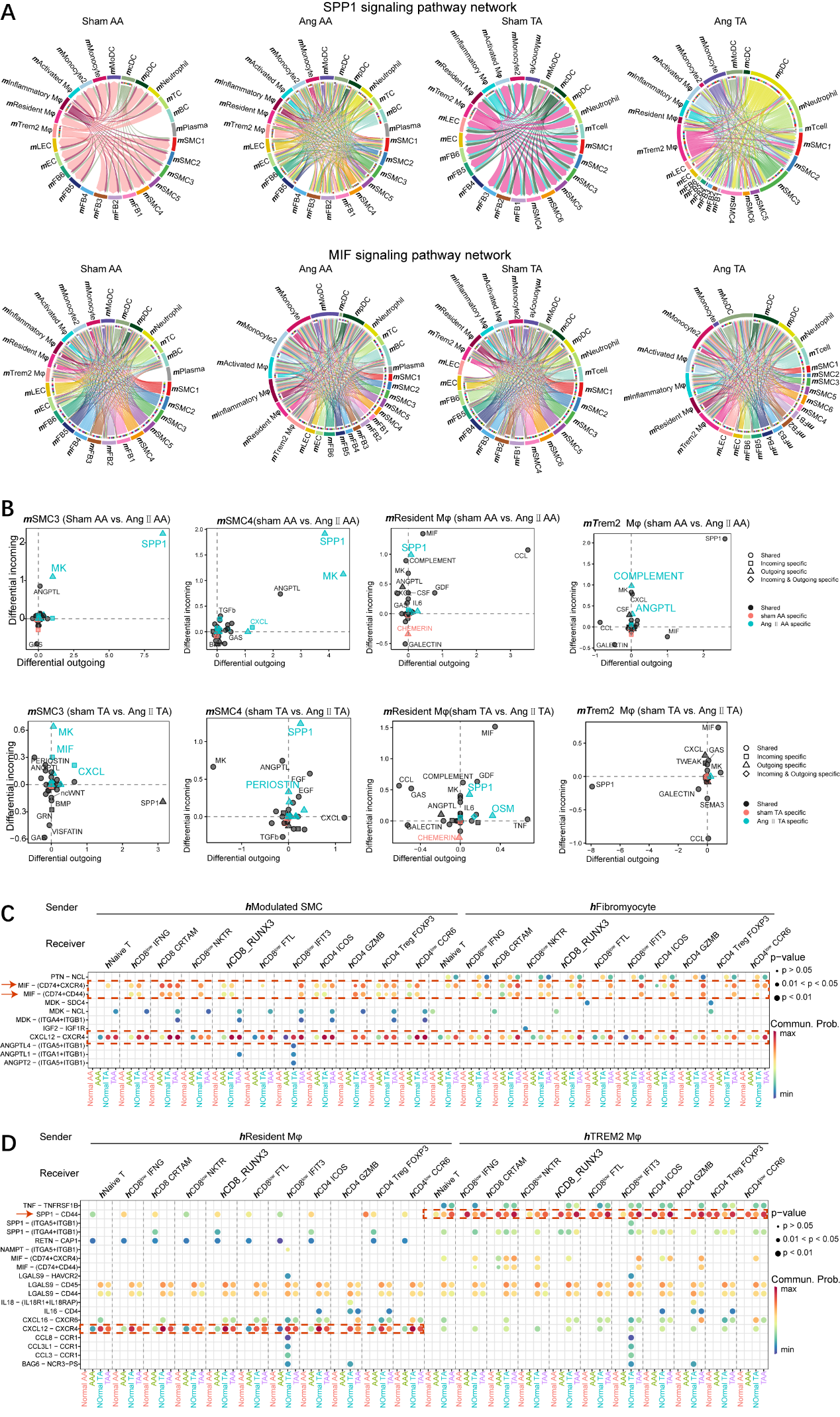


**Figure S7：MIF and SPP1 Signaling Pathways Commonly Altered Among Different Species**

1. Chord diagram of SPP1 (upper) and MIF (bottom) signaling network in mouse samples (sham AA, Ang Ⅱ AA, sham TA and Ang Ⅱ TA).
2. Signaling changes of cell subpopulations (***m***SMC3, ***m***SMC4, ***m***Resident Mφ and ***m***Trem2 Mφ) in Ang Ⅱ AA (upper) and Ang Ⅱ TA (bottom) compared to respective control group.
3. Comparison of the significant ligand-receptor pairs between normal AA, AAA, normal TA and TAA, which contribute to the signaling from ***h***Modulated SMC and ***h***Fibromyocte to all T cell subsets. Dot color reflects communication probabilities and dot size represents computed p-values. Empty space means the communication probability is zero. p-values are computed from one-sided permutation test.
4. Comparison of the significant ligand-receptor pairs between normal AA, AAA, normal TA and TAA, which contribute to the signaling from ***h***Resident Mφ and ***h***TREM2 Mφ to all T cell subsets.
